# ATP6AP2, a regulator of LRP6/β-catenin protein trafficking, promotes Wnt/β-catenin signaling and bone formation in a cell type dependent manner

**DOI:** 10.1038/s41413-024-00335-7

**Published:** 2024-05-29

**Authors:** Lei Xiong, Hao-Han Guo, Jin-Xiu Pan, Xiao Ren, Daehoon Lee, Li Chen, Lin Mei, Wen-Cheng Xiong

**Affiliations:** 1 Department of Neurosciences, School of Medicine, Case Western Reserve University, Cleveland, OH 44106 USA; 2 Louis Stoke VA Medical Center, Cleveland, OH 44106 USA

**Keywords:** Bone, Bone quality and biomechanics

## Abstract

Wnt/β-catenin signaling is critical for various cellular processes in multiple cell types, including osteoblast (OB) differentiation and function. Exactly how Wnt/β-catenin signaling is regulated in OBs remain elusive. ATP6AP2, an accessory subunit of V-ATPase, plays important roles in multiple cell types/organs and multiple signaling pathways. However, little is known whether and how ATP6AP2 in OBs regulates Wnt/β-catenin signaling and bone formation. Here we provide evidence for ATP6AP2 in the OB-lineage cells to promote OB-mediated bone formation and bone homeostasis selectively in the trabecular bone regions. Conditionally knocking out (CKO) ATP6AP2 in the OB-lineage cells (*Atp6ap2*^*Ocn-Cre*^) reduced trabecular, but not cortical, bone formation and bone mass. Proteomic and cellular biochemical studies revealed that LRP6 and N-cadherin were reduced in *ATP6AP2-KO* BMSCs and OBs, but not osteocytes. Additional in vitro and in vivo studies revealed impaired β-catenin signaling in *ATP6AP2-KO* BMSCs and OBs, but not osteocytes, under both basal and Wnt stimulated conditions, although LRP5 was decreased in *ATP6AP2-KO* osteocytes, but not BMSCs. Further cell biological studies uncovered that osteoblastic ATP6AP2 is not required for Wnt3a suppression of β-catenin phosphorylation, but necessary for LRP6/β-catenin and N-cadherin/β-catenin protein complex distribution at the cell membrane, thus preventing their degradation. Expression of active β-catenin diminished the OB differentiation deficit in *ATP6AP2-KO* BMSCs. Taken together, these results support the view for ATP6AP2 as a critical regulator of both LRP6 and N-cadherin protein trafficking and stability, and thus regulating β-catenin levels, demonstrating an un-recognized function of osteoblastic ATP6AP2 in promoting Wnt/LRP6/β-catenin signaling and trabecular bone formation.

## Introduction

Bone remodeling is tightly regulated by bone-forming osteoblasts (OBs) and bone-resorbing osteoclasts (OCs).^1,2^ OBs are differentiated from bone marrow stromal cells (BMSCs) or mesenchymal progenitor cells, whereas OCs are derived from hematopoietic bone marrow macrophages or myeloid monocytes (BMMs). Healthy bone mass is maintained by balanced activity of OBs and OCs. The imbalance of bone formation and resorption could result in bone loss such as osteoporosis or high-bone mass disease such as sclerosteosis and Van Buchem disease.^1,3^

Bone remodeling and bone mass homeostasis are regulated by multiple signaling pathways, including Wnt/β-catenin signaling. Binding of Wnt ligands (Wnts) to a dual-receptor complex of frizzled (Fzd) and LRP5/6 leads to accumulation of cytoplasmic β-catenin and translocation of β-catenin into the nucleus, which regulates gene expressions for OB mediated bone formation as well as the OC-regulated bone resorption. In particularly, this pathway is required for commitment of mesenchymal stem cells to the OB lineage, and OB precursor cell proliferation and differentiation.^1–3^ Notice that loss-of-function of LRP6 in humans leads to multiple disorders, including triglycerides, hypertension, diabetes, and osteoporosis.^4^ Loss-of-function of LRP5 is found to cause osteoporosis-pseudoglioma syndrome,^5,6^ while gain-of-function mutations in LRP5 induced high bone mass.^7,8^ These observations provide compelling evidence for LRP5/6’s function in bone mass homeostasis.

ATP6AP2 (ATPase H^+^ transporting accessory protein 2), also called PRR (pro-renin receptor), plays important roles in multiple signaling pathways.^9–13^ It is initially identified as PRR, because it binds to pro-renin and renin, and induces the conversion of angiotensinogen to angiotensin I.^12^ Later, ATP6AP2 is identified as an accessory subunit of V-ATPase, a proton pump containing macromolecular complex, regulating the V-ATPase protein complex assembly and activity.^14,15^ Recently, it is recognized as a critical regulator of Wnt/β-catenin signaling.^16,17^ ATP6AP2 interacts with Wnt receptors, LRP6 and frizzled, in HEK293 cells, *Xenopus laevis tadpoles*, and *Drosophila*.^16,17^ Suppression of ATP6AP2 in HEK293 cells or in *Drosophila* reduces Wnt canonical (β-catenin) and non-canonical (PCP, planar cell polarity) signaling.^16,17^ Meanwhile, ATP6AP2 is a direct downstream target of Wnt/β-catenin signaling.^18^ As such, ATP6AP2 could amplify Wnt/β-catenin signaling via a self-perpetuating cycle. Whereas ATP6AP2 is believed to be a critical regulator for Wnt/β-catenin signaling, exactly how it regulates Wnt/β-catenin signaling and function remains elusive.

Functionally, ATP6AP2 plays important roles in multiple organs and cell types; and dysregulation of ATP6AP2 is implicated in the pathogenesis of numerous diseases, such as hypertension, pre-eclampsia, diabetic microangiopathy, acute kidney injury, cardiovascular disease, cancer, obesity, mental disorders (e.g., depression and post-traumatic stress disorder), and neurodegenerative diseases (e.g., Parkinson disease and Alzheimer disease).^9,19–31^ Notice that ATP6AP2 is abundantly expressed in bone cells, including OBs and OCs. However, its functions in bone or bone disorders remain to be investigated.

Here, we investigated ATP6AP2’s function in OBs by use of mouse with selective depletion of ATP6AP2 in osteoblast (OB)-lineage cells (*Atp6ap2*^*Ocn-Cre*^). *Atp6ap2*^*Ocn-Cre*^ mice exhibit reduced trabecular, but not cortical, bone density, which was tightly associated with the impairment in OB-mediated bone formation. We then carried out proteomics analysis in *ATP6AP2-KO* OB-lineage cells (compared with that of control cells) and identified LRP6, whose cell surface distribution is decreased in *ATP6AP2-KO* OB-lineage cells in culture. Further studies suggest that the reduced trabecular bone formation is likely due to an impairment in Wnt/LRP6/β-catenin signaling in *ATP6AP2-KO* BMSCs, OBs, but not osteocyte; and expression of active β-catenin diminished the OB differentiation deficit in *Atp6ap2*^*Ocn-Cre*^ BMSCs. Further mechanistic studies suggest that the osteoblastic ATP6AP2 stabilizes β-catenin, independent on β-catenin phosphorylation/dephosphorylation, but likely by promoting LRP6/β-catenin and/or N-cadherin/β-catenin protein complex trafficking to the cell surface, and thus preventing their degradation. Taken together, these results suggest a bone region or cell type selective function of ATP6AP2 in promoting Wnt/β-catenin signaling in OBs at the trabecular bone region, identifying ATP6AP2 as a key regulator for LRP6/β-catenin and N-cadherin/β-catenin trafficking and stability.

## Results

### Decreased trabecular, but increased cortical, bone-mass in *Atp6ap2*^*Ocn-Cre*^ mice

To investigate ATP6AP2’s function in bone, we generated OB-selective ATP6AP2 conditional knockout (CKO) mice, *Atp6ap2*^*Ocn-Cre*^, by crossing *Atp6ap2*^*flox/X*^ with *osteocalcin (Ocn)-Cre* mice (Fig. S1a), whose cre expression is under the control of the human bone gamma carboxyglutamate (BGLAP) promoter/enhancer, and thus largely in the OB-lineage cells, including bone marrow stromal cells (BMSCs), OBs, and osteocytes.^2,10,32,33^ As expected, ATP6AP2 protein levels were selectively reduced in the OB-lineage, but not the OC-lineage, cells in *Atp6ap2*^*Ocn-cre*^ mice, as compared with those of littermate control mice (*Ocn-Cre* mice) (Fig. S1b–e). *Atp6ap2*^*Ocn-cre*^ mice showed reduced body size and body weight, started as early as P15 (postnatal day 15) (Fig. S1f, g), implicating a deficit in mouse skeleton development.

**Fig. 1:**
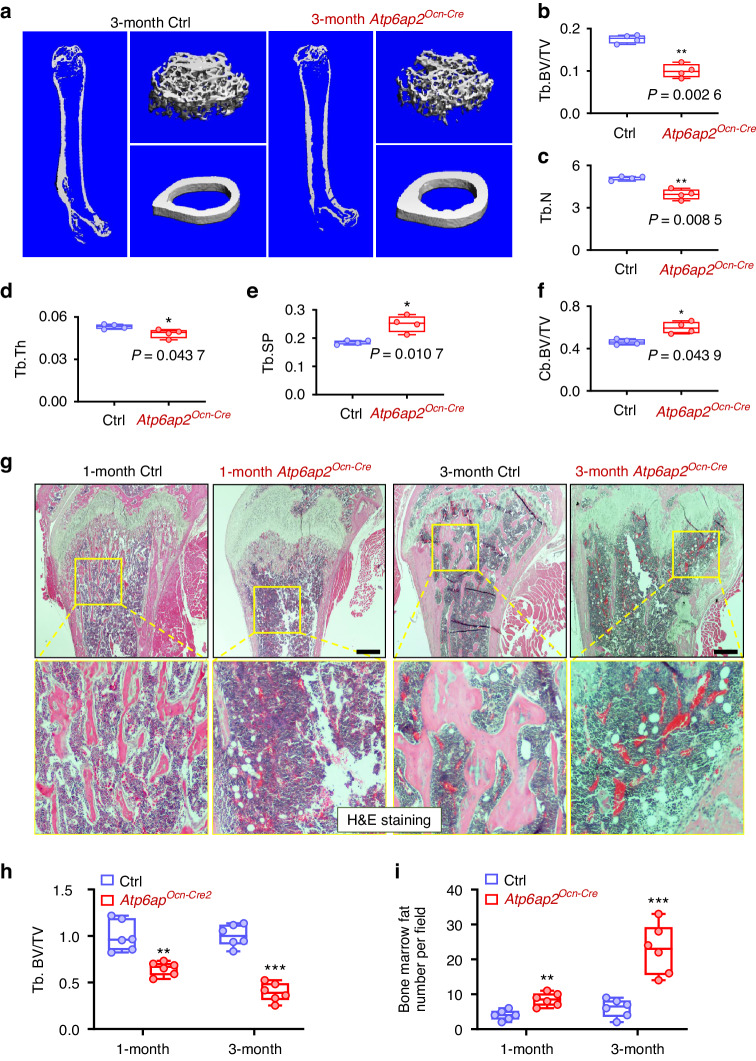
Reduced trabecular, but increased cortical, bone-mass in *Atp6ap2*^*Ocn-Cre*^ mice. **a** Representative μCT 3D images of femurs from 3-month male ctrl and *Atp6ap2*^*Ocn-Cre*^ mice littermates. **b–f** Quantification analyses of trabecular bone (Tb) volumes over total volumes (BV/TV), Tb number (Tb.N), Tb thickness (Tb.TH), Tb space (Tb. SP) and cortical bone (Cb) volumes over total volumes (BV/TV) by direct model of μCT analysis. **g** Representative images of H&E stained femur sections from male ctrl and *Atp6ap2*^*Ocn-Cre*^ mice at ages of 1-month and 3-month. Bar, 150 μm. **h**, **i** Quantification analyses of trabecular bone (Tb) volumes over total volumes (BV/TV) and bone marrow fat number in (**g**). Data in (**b**–**f**), (**h**) and (**i**) are shown as box plots together with individual data points, and whiskers indicate minimum to maximum (*n* = 4 or 6 male mice of each genotype). *P* values obtained by unpaired two-tailed *t* test. *P < 0.05. **P < 0.01. ***P < 0.001

We further examined femur structures in control and *Atp6ap2*^*Ocn-cre*^ mice by microcomputer tomographic (μCT) analysis. Decreases in trabecular bone volumes over total volumes (BV/TV), trabecular bone number (Tb. N) and trabecular bone thickness (Tb.TH), and increases in trabecular bone space (Tb. Sp), and cortical bone volumes/total volumes (Cb, BV/TV) were all detected in *Atp6ap2*^*Ocn-Cre*^ mice (3-month), as compared with those of control littermates (*Ocn-Cre* mice) (Fig. 1a–f). The trabecular bone deficit was further verified by H&E staining analysis (Fig. 1g–i). H&E staining analysis of the femur bone sections showed a marked reduction in the trabecular bone-mass and an increase in bone marrow fat as early as 1-month in *Atp6ap2*^*Ocn-cre*^ mice (Fig. 1g–i). These results demonstrated a trabecular, but not cortical, bone-loss, and suggest the necessity of osteoblastic ATP6AP2 in trabecular bone homeostasis.

### Enhanced OC formation and bone resorption in *Atp6ap2*^*Ocn-Cre*^ trabecular and cortical bone regions

The bone-mass is controlled by both bone formation and bone resorption.^34,35^ We thus examined osteoclastic bone resorption in control and *Atp6ap2*^*Ocn-Cre*^ mice. Measuring serum levels of deoxy-pyridinoline (PYD, a marker for bone resorption) demonstrated elevated PYD levels, suggesting an increased bone resorptive activity in the mutant mice at the ages of 1-month and 3-month (Fig. S2a). We then carried out TRAP staining analysis of OCs, which showed an increase in the number per unit of both trabecular and cortical bone surface in both 1-month and 3-month *Atp6ap2*^*Ocn-Cre*^ mice (Fig. S2b–d). We next asked whether ATP6AP2 regulates OC formation and resorptive activity in vitro by examining M-CSF- and RANKL-induced OC differentiation from BMMs derived from *Atp6ap2*^*Ocn-Cre*^ and control mice. More TRAP^+^ MNCs (multi-nuclei cells) were detected in BMM cultures from *Atp6ap2*^*Ocn-Cre*^ mice than that of the control BMMs on day 5 of RANKL treatment (Fig. S2e, f), implicating that more OC progenitors were present in the cultured BMMs from *Atp6ap2*^*Ocn-Cre*^ mice. To determine whether these MNCs have resorptive function, we cultured these cells on coverslips coated with inorganic hydroxyapatite matrix. An increase in the resorptive pit-like area was detected in the mutant MNCs or OCs, as compared with that of control cells (Fig. S2e, g). Together, these results uncover a role of osteoblastic ATP6AP2 in preventing hyper-active OC formation and activation in both trabecular and cortical bone regions, unlike the selective trabecular bone loss, but increase of cortical bone mass, in *Atp6ap2*^*Ocn-Cre*^ mice, and implicating additional pathological mechanism(s) to underlie the bone phenotypes in the mutant mice.

### Reduced trabecular, but elevated cortical, bone formation in *Atp6ap2*^*Ocn-Cre*^ mice

**Fig. 2:**
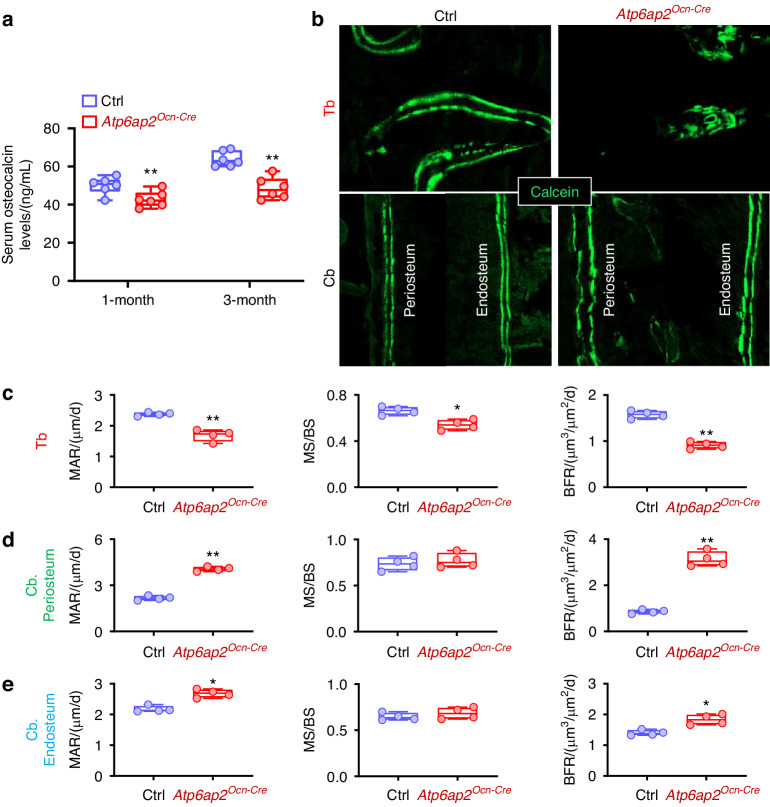
Decreased trabecular, but increased cortical, bone formation in *Atp6ap2*^*Ocn-Cre*^ mice. **a** The serum osteocalcin levels in 1-month and 3-month male ctrl and *Atp6ap2*^*Ocn-Cre*^ mice were measured by ELISA. **b** Representative images of histologic sections showing calcein labeling of trabecular, periosteum and endosteum bone regions (diaphysis region at the same distance from the growth plate, close to metaphysis) in femur of male ctrl and *Atp6ap2*^*Ocn-Cre*^ mice at age of 1-month. **c–e** MAR (mineral apposition rate), MS (mineral surface) / BS (bone surface), and BFR (bone formation rate) are presented. Data in (**a**) and (**c**–**e**) are shown as box plots together with individual data points, and whiskers indicate minimum to maximum (*n* = 4 to 6 animals per genotype). *P* values obtained by unpaired two-tailed *t* test. *P < 0.05. **P < 0.01

We then examined bone formation in the control and *Atp6ap2*^*Ocn-Cre*^ mice. First, measuring serum levels of osteocalcin, a marker for bone formation, by ELISA showed decreases in both 1-month and 3-month *Atp6ap2*^*Ocn-Cre*^ mice, as compared with those of littermate control mice (Fig. 2a). Second, examining bone formation in both trabecular and cortical bones of the control and mutant mice by injecting fluorochrome-labeled calcein green into 1-month mice twice at a 5-days interval showed decreases in mineral apposition rate (MAR), mineral surface/bone surface (MS/BS), and bone formation rate (BFR) selective in trabecular bone region (Fig. 2b, c). In contrast, in the mutant periosteum and endosteum bone regions, the MAR, MS/BS, and BFR were all increased, as compared with those of control mice (Fig. 2b, d, e). These results thus demonstrate an association of the trabecular/cortical bone phenotypes with the altered bone formation rate in *Atp6ap2*^*Ocn-Cre*^ mice. Third, using in vitro OB differentiation assay, BMSCs from *Atp6ap2*^*Ocn-cre*^ mice formed less ALP^+^ OBs and lower calcified bone matrix viewed by Alizarin Red S staining than those of OBs derived from control BMSCs (Fig. S3a–c). In addition, the transcript levels of Runx2 (Runt-related transcription factor 2) and Osx (Osterix), both transcription factors that play important roles in OB differentiation and bone formation,^36^ were also significantly reduced in *Atp6ap2-KO* BMSCs during osteogenic differentiation (Fig. S3d). These results demonstrate a reduced OB-genesis and function in the mutant mice, suggest a cell autonomous role of ATP6AP2 in this event, and implicate the bone region selective alteration of the bone formation rate to underlie the regional specific bone phenotypes in *Atp6ap2*^*Ocn-Cre*^ mice.

### Diminished LRP6 in *ATP6AP2-KO* OBs

**Fig. 3:**
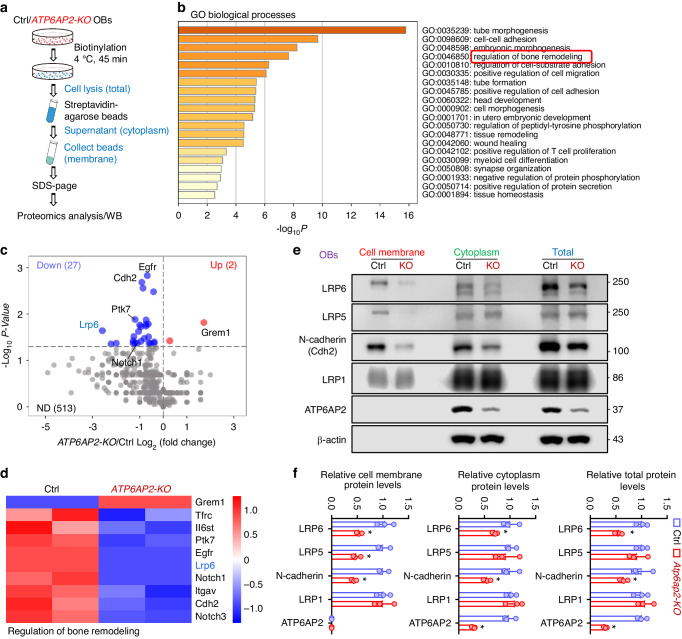
Decreased LRP6 and N-cadherin surface distribution and total protein levels in *ATP6AP2-KO* OBs. **a** Schematic diagram of quantitative proteomic analysis. Proteomic data were obtained from surface proteins of ctrl and *ATP6AP2-KO* OBs. **b** The gene ontology (GO) Biological Processes analysis shows the top 20 significant enrichment terms represented by biological processes, with longer column representing more significant enrichment. Many proteins that regulate bone remodeling were involved. **c** Volcano plots of differentially expressed proteins in the cell surface of ctrl and *ATP6AP2-KO* OBs. The up-regulated proteins were marked in red, and the down-regulated proteins were indicated in blue (*P* < 0.05). **d** Heat map protein expression z-scores computed for the 10 proteins that were involved in regulation of bone remodeling. **e**, **f** Western blot analyses of lysates of total, cytoplasm and cell surface fractions of ctrl and *ATP6AP2-KO* OBs. Quantification of the data was shown in (**f**). Data in (**f**) are presented as mean ± SD (*n* = 3). *P* values obtained by unpaired two-tailed *t* test. *P < 0.05

To investigate how osteoblastic ATP6AP2 regulates trabecular bone formation, we carried out proteomic analysis using liquid chromatography-tandem mass spectrometry to screen for altered plasma membrane proteins in primary cultured *ATP6AP2-KO* Ocn-Cre^+^ OBs (Fig. 3a). Both Ctrl (Ocn-Cre) and *ATP6AP2-KO* OBs were incubated with NHS-biotin for 45 min at 4 °C to label cell surface membrane proteins. The biotin labeled surface proteins were pulled down by streptavidin-agarose beads and subjected to proteomic analysis and Western blot analysis. Among a total of 542 proteins identified, 27 proteins were down-regulated, and 2 proteins was up-regulated in *ATP6AP2-KO* OBs, as compared with those of ctrl (Fig. 3b, c). Gene ontology (GO) biological processes analysis showed several biological processes, including tube morphogenesis, cell-cell adhesion, and regulation of bone remodeling that were altered by *ATP6AP2-KO* (Fig. 3b). Notice that the proteins in the regulation of bone remodeling pathway include the Wnt/β-catenin pathway proteins, such as Lrp6, Cdh2 (N-Cadherin) and Ptk7 (Fig. 3d). Given the reports that LRP6 is a receptor for Wnt/β-catenin signaling,^1,37^ an essential pathway for osteoblastic bone formation, and N-cadherin/β-catenin not only controls cell-cell adhesion, but also proliferation, differentiation, and survival of mesenchymal cells,^38^ we further examined LRP6, its homolog LRP5, as well as N-cadherin’s cell surface levels in control and *ATP6AP2-KO* OB-lineage cells. Western blot analysis of lysates of subcellular fractions of OBs showed that the cell surface levels of LRP6, LRP5 and N-cadherin were lower in *ATP6AP2-KO* OB-progenitors than those of controls (Fig. 3e, f), suggesting ATP6AP2’s function in promoting their surface targeting. The cytoplastic and total levels of LRP6 and N-cadherin, but not LRP5, were lower in the mutant OBs (Fig. 3e, f). The reductions of these membrane proteins appeared to be selective, as the LRP1 level was un-changed. The mRNA level of Lrp6 in the mutant OBs was comparable to that of control OBs (Fig. S4a), suggesting that ATP6AP2 increases LRP6 at the post-transcriptional level.

### Attenuation of LRP6/β-catenin signaling in *ATP6AP2-KO* OBs, but not osteocytes, in culture and in vivo

**Fig. 4:**
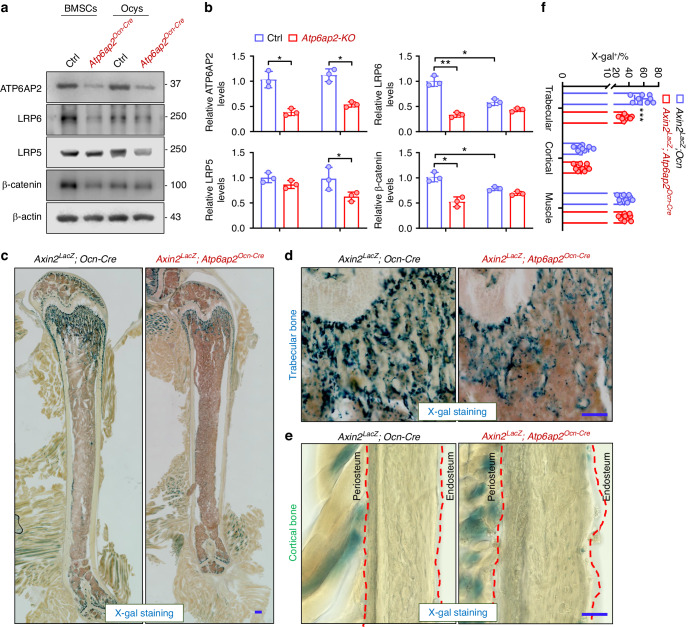
Impairment of Wnt/β-catenin signaling selectively in the OBs of trabecular bone, but not osteocytes of cortical bone, of *Atp6ap2*^*Ocn-Cre*^ mice. **a**, **b** Decreased LRP6 and β-catenin expression in BMSCs, buy not Ocys, derived from 3-month *Atp6ap2*^*Ocn-Cre*^ mice by Western blots. **a** Representative blots; and (**b**). Quantification analysis. **c–f** Selectively decreased β-catenin signaling in the trabecular bone of *Atp6ap2*^*Ocn-Cre*^ mice at 1-month, by *Axin2*^*LacZ*^ reporter activity. Ctrl and *Atp6ap2*^*Ocn-Cre*^ mice are crossed with *Axin2*^*LacZ*^ reporter line and β-galactosidase activity is revealed by X-gal staining. Representative images were shown in (**c**–**e**). Bar, 50 μm. Quantification analysis as mean ± SD was shown in (**f**). Data in (**b**) and (**f**) are presented as mean ± SD (*n* = 3 or 9). *P* values obtained by unpaired two-tailed *t* test. *P < 0.05. **P < 0.01. ***P < 0.001, significant difference

The OB-lineage cells include BMSCs, OBs, and osteocytes.^39–41^ To address whether ATP6AP2 regulates LRP6 and LRP5 proteins in these OB-lineage cells, we further examined LRP6 and LRP5 levels in lysates from BMSCs and osteocytes (Ocys) derived from control and *Atp6ap2*^*Ocn-cre*^ mice by Western blot. Notice that LRP6 appeared to be less in Ocys than that of BMSCs (Fig. 4a, b); and β-catenin, but not ATP6AP2, was also lower in Ocys than that of BMSCs (Fig. 4a, b), implicating a more critical role of LRP6/β-catenin signaling in the trabecular bone region. Interestingly, both LRP6 and β-catenin were decreased in the mutant BMSCs, but not Ocys, as compared with those of control cells (Fig. 4a, b). In contrast, the total level of LRP5 was selectively reduced in the mutant Ocys, but not BMSCs, implicate a differential regulation of LRP5 and LRP6 by ATP6AP2 in a cell subtype selective manner. Together, these results suggest that ATP6AP2 selectively regulate LRP6 and β-catenin levels in the early stage of OB-lineage cells (e.g., BMSCs and OBs) but regulates LRP5 in the later stage (e.g., Ocys), providing additional evidence for ATP6AP2’s function in regulating Wnt/β-catenin signaling in a cell type or bone region selective manner.

To test above view in vivo, we crossed *Axin2*^*LacZ*^ mice, a Wnt/β-catenin signaling LacZ reporter mouse line, with *Atp6ap2*^*Ocn-cre*^ to generate *Axin2*^*LacZ*^; *Atp6ap2*^*Ocn-cre*^ mice. X-gal staining analysis revealed abundant β-galactosidase activity in the trabecular region of the ctrl mice (*Axin2*^*LacZ*^; *Ocn-Cre*), which was marked reduced in the mutant mice (Fig. 4c, d, f). In contract, β-galactosidase activity in the skeletal muscle, where ATP6AP2 has not been knocked out, showed no significant changes in the mutant mice compared with the ctrl mice, supporting the view for ATP6AP2 as a positive regulator of Wnt/β-catenin signaling in vivo.^16,17^ Notice that in the cortical bone diaphysis regions of both control and mutant mice, the β-galactosidase activity was weakly detectable, but comparable between control and mutant mice (Fig. 4c, e, f). These results thus provide in vivo evidence that ATP6AP2 is required for Wnt/β-catenin signaling in the OB-lineage cells (OBs) at the trabecular bone region, but not osteocytes in the cortical region.

### Impairments in Wnt3a induced β-catenin signaling and OB genesis in cultured BMSCs from *Atp6ap2*^*Ocn-Cre*^ mice

**Fig. 5:**
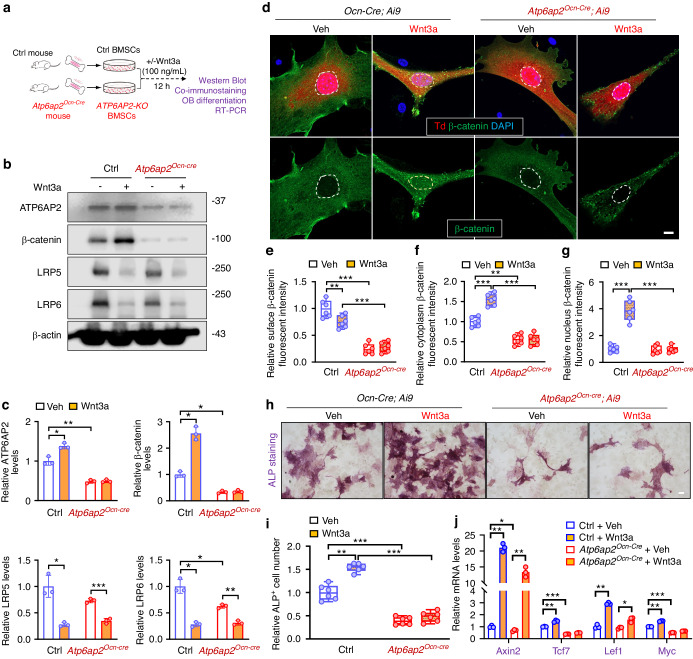
Attenuation of Wnt/β-catenin signaling in *Atp6ap2*^*Ocn-Cre*^ BMSCs. **a** Experimental strategy. Cells were treated with 100 ng/mL Wnt3a for 12 h as indicated. **b**, **c** Western blot analysis of ATP6AP2, total-β-catenin, LRP5 and LRP6 expression in BMSCs from indicated mice. Quantitative data were shown in (**c**). **d–g** Primary cultured control and *ATP6AP2-KO* BMSCs were treated with 100 ng/mL Wnt3a for 6 h. Fluorescence microscopy was undertaken to monitor β-catenin. Bar, 10 µm. Quantification analysis was shown in (**e**–**g**). **h**, **i** ALP staining analysis of cultured BMSCs in the presence of veh or Wnt3a. Bar, 20 µm. Quantitative analysis of the average ALP^+^ cell number was shown in (**i**). **j** Real-time PCR analysis of gene expressions in BMSCs derived from control and *ATP6AP2-KO* mice after wnt3a treatment (12 h). Data in (**c**), (**e**–**g**) and (**i**–**j**) are presented as mean ± SD (*n* = 3 or 6). *P* values obtained by two-way ANOVA followed by Bonferroni post hoc test. *P < 0.05. **P < 0.01. ***P < 0.001, significant difference

LRP6, one of receptors for Wnts, mediates accumulation and nuclear translocation of β-catenin, and regulates a series of target genes expression and multiple cellular processes, including OB genesis.^1–3^ We thus asked whether ATP6AP2 is necessary for Wnt3a induced β-catenin signaling and OB formation. Primary cultured BMSCs derived from ctrl and *Atp6ap2*^*Ocn-Cre*^ mice were treated with recombinant Wnt3a protein for overnight (Fig. 5a). Western blot results showed that Wnt3a treatment increased the β-catenin level in the control BMSCs (Fig. 5b, c). However, in the *ATP6AP2-KO* BMSCs, Wnt3a failed to induce the β-catenin level, in addition to the lower β-catenin and LRP6 in the mutant cells under basal condition (Fig. 5b, c). Notice that wnt3a treatment enhanced ATP6AP2 but reduced Lrp6/Lrp5 protein levels and mRNA levels (Fig. 5b, c, Fig. S4a–c). This may be a kind of negative feedback on Wnt3a treatment. These results suggest a necessity of osteoblastic ATP6AP2 in maintaining both basal and Wnt3a induced β-catenin levels. Moreover, immunofluorescence staining analysis showed that Wnt3a induced the nuclear localization of β-catenin in control, but not *ATP6AP2-KO*, BMSCs (Fig. 5d–g), compared with control, *ATP6AP2-KO* BMSCs also showed decreased surface and cytoplasm levels of β-catenin (Fig. 5d–g), providing evidence for a requirement of ATP6AP2 in Wnt3a induced β-catenin nuclear distribution.

We then asked whether ATP6AP2 is necessary for Wnt3a induced OB-genesis. BMSCs from control and *Atp6ap2*^*Ocn-Cre*^ mice were induced for OB-differentiation in the presence or absence of Wnt3a. As shown in Fig. 5h, i, Wnt3a significantly enhanced ALP^+^ OBs in control BMSCs, but little to no induction in *ATP6AP2-KO* BMSCs (Fig. 5h, i), supporting the view for the requirement of ATP6AP2 in this event. We further tested this view by examining the expression of Wnt/β-catenin target genes, including Axin2, Tcf7, Lef1 and Myc. As shown in Fig. 5j, RT-PCR analysis showed increased expression of all of these β-catenin target genes in the control cells; but in *ATP6AP2-KO* cells, the inductions in Tcf7 and Myc, but not Axin2 or Lef1, were abolished (Fig. 5j), suggesting a selective role of ATP6AP2 in regulating Wnt3a/β-catenin target gene expression for OB-differentiation.

Notice that ATP6AP2-deficiency in BMSCs also impaired the expression of osteoprotegerin (OPG), thus increased the ratio of receptor activator of nuclear kappa B ligand (Rankl) over OPG, which may underlie the increased OC formation in *ATP6AP2-KO* mice (Fig. S4d). Wnt3a could up-regulate the expression of OPG thus reduce the ratio of Rankl/OPG in the ctrl, but not in the *ATP6AP2-KO* group (Fig. S4d). These results suggested that the impairment of Wnt/β-catenin signaling in *Atp6ap2*^*Ocn-Cre*^ BMSCs may be responsible for not only the reduced OB genesis and OB mediated bone formation, but also the OC-genesis and OC mediated bone resorption.

### Independent on ATP6AP2 for Wnt3a suppression of β-catenin phosphorylation

**Fig. 6:**
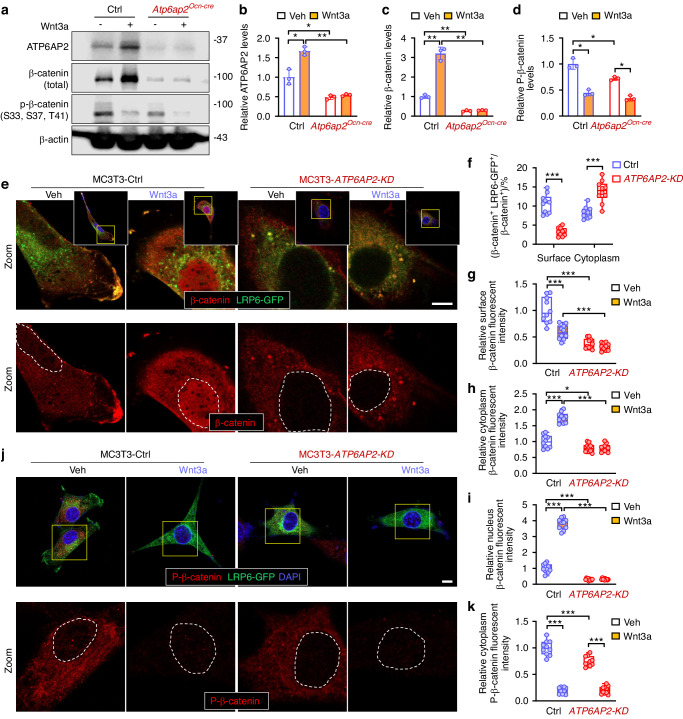
Wnt3a inhibition of phosphor-β-catenin in ATP6AP2 independent manner. **a–d** Primary cultured control and *ATP6AP2-KO* BMSCs were treated with 100 ng/mL Wnt3a for 12 h. Western blotting analysis of ATP6AP2, β-catenin and phosphor-β-catenin (Ser33/37, Thr41) were shown in (**a**). β-actin was used as the loading controls. Quantification analyses were shown in (**b**-**d**). **e–k** Immunostaining analysis of β-catenin (**e**) and phosphor-β-catenin (**j**) in ctrl and *ATP6AP2-KD* MC3T3 cells transfected with the LRP6-eGFP. Cells were treated with 100 ng/mL Wnt3a for 6 h. Bar, 10 µm. Images marked with yellow squares were amplified and shown below. Nuclei were marked with white dashed lines. Quantification analyses were shown in (**f**, **g**, **h**, **i**, **k**). Data in (**b**–**d**) are presented as mean ± SD (*n* = 3). Data in (**f**–**i**) and (**k**) are shown as box plots together with individual data points, and whiskers indicate minimum to maximum (*n* = 10). *P* values obtained by two-way ANOVA followed by Bonferroni post hoc test. *P < 0.05. **P < 0.01. ***P < 0.001

To investigate how ATP6AP2 increases β-catenin expression in both basal and Wnt3a stimulated conditions, we first examined β-catenin’s mRNA levels in control and *ATP6AP2 KO* BMSCs. The mRNA levels of β-catenin in *ATP6AP2-KO* cells showed no significantly change compared with the ctrl, indicating that ATP6AP2 mainly regulates the protein level of β-catenin (Fig. S4e). Second, we examined β-catenin phosphorylation, a process mediated by GSK3β, suppressed by Wnt3a, and critical for β-catenin degradation,^1,3^ in control and *ATP6AP2 KO* BMSCs treated with or without Wnt3a. Western blot analysis showed that the phosphorylations of β-catenin, recognized by the phosphor-Ser33/37/Thr41 antibodies, were indeed decreased by Wnt3a in control cells (Fig. 6a–d). Unexpectedly, such a Wnt3a suppression of phosphor-β-catenin was also detectable in *ATP6AP2-KO* cells (Fig. 6a–d). Similar to these results were the observations by co-immunostaining analyses, which showed marked decrease of phosphor-β-catenin in both control and *ATP6AP2-KD* MCT3T3 cells in response to Wnt3a, while the total β-catenin, in particular the nuclear and cytoplasm β-catenin levels were induced by Wnt3a in control, but not *ATP6AP2-KD* cells (Fig. 6e–k). We further examined the ubiquitination of β-catenin and found that Wnt3a decreased ubiquitin conjugated β-catenin in ctrl, but not in *ATP6AP2-KD* MC3T3 cells (Fig. S5), suggesting a role of ATP6AP2 in Wnt3a suppression of β-catenin’s ubiquitination or ubiquitin mediated proteosome degradation. These results thus suggest an independence of ATP6AP2 in Wnt3a suppression of β-catenin phosphorylation, implicating β-catenin phosphorylation/dephosphorylation independent mechanism(s) to be involved in ATP6AP2 stabilizing β-catenin.

### Requirement of ATP6AP2 for LRP6/β-catenin targeting to the cell surface and preventing LRP6 distribution in the lysosomes

Notice that both LRP6 and β-catenin were lower in *ATP6AP2-KO* cells (Fig. 5b, c); and LRP6 interacts with ATP6AP2.^17^ In light of these observations, we speculate that ATP6AP2 may stabilize β-catenin via LRP6. To test this view, we first examined whether the distribution of β-catenin and LRP6 were altered in *ATP6AP2-KD* MC3T3 cells. LRP6-GFP was transfected into ctrl and *ATP6AP2-KD* MC3T3 cell lines. As shown in Fig. 6e, f, the β-catenin was co-localized with LRP6 largely on the cell membrane. However, in the *ATP6AP2-KD* cells, the β-catenin was significantly decreased in the cell membrane, and it was mainly co-localized with LRP6 in the cytoplasm (Fig. 6e–i). Additionally, *ATP6AP2-KD* significantly reduced the nucleus distribution of β-catenin, as compared with that in ctrls (Fig. 6e–i).

To understand how ATP6AP2 promotes LRP6 surface targeting, we examined ATP6AP2 and LRP6 interaction and mapped the binding domain in ATP6AP2. ATP6AP2 and its deletion mutants were co-transfected with LRP6-GFP into MC3T3. As shown in Fig. S6a–c, ATP6AP2 appeared to form a complex with LRP6 via its ECD domain. Interestingly, the ICD domain, also called M8.9, which binds to the V-ATPase complex proteins and promotes V-ATPase complex assemble and activation,^42^ was not co-localized with LRP6 (Fig. S6a–c), implicating that ATP6AP2 may interact with LRP6 independent on its association with V-ATPase.

In addition to the reduced LRP6 surface targeting in *ATP6AP2-KD* MC3T3 cells, LRP6 in EEA1^+^ endosomes were decreased, but in Lamp1^+^ lysosomes were increased (Fig. S6d, e). We then further examined LRP6’s half-life. Indeed, a shorter half-life of LRP6 was detected in *ATP6AP2-KD* MC3T3 cells (Fig. S6f, g). These results suggest that ATP6AP2 may increase LRP6 stability by promoting its recycle from endosomes to the cell surface; and thus prevent its degradation (Fig. S6h).

### Requirement of ATP6AP2 for N-cadherin/β-catenin protein complex distribution in the cell surface

**Fig. 7:**
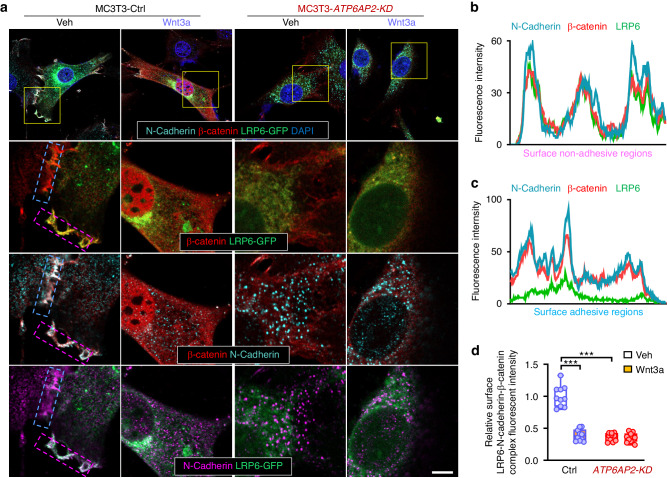
Requirement of ATP6AP2 for N-cadherin/β-catenin protein complex distribution in the cell surface. **a** Immunostaining analysis of N-cadherin and β-catenin in ctrl and *ATP6AP2-KD* MC3T3 cells transfected with the Lrp6-eGFP. Cells were treated with 100 ng/mL Wnt3a for 6 h. Bar, 10 µm. Images marked with yellow squares were amplified and shown below. The blue dotted box marked surface adhesive regions, and the purple dotted box marked surface non-adhesive regions. **b**, **c** Quantification analyses of LRP6, N-cadherin, and β-catenin fluorescence distribution in the marked regions. **d** Quantification analyses of the LRP6-N-cadherin-β-catenin complex fluorescence intensity in the surface of cells. Data in (**d**) are shown as box plots together with individual data points, and whiskers indicate minimum to maximum (*n* = 10). *P* values obtained by two-way ANOVA followed by Bonferroni post hoc test. ***P < 0.001

In addition to LRP6, our proteomics analysis and western blot analysis showed reduced cell surface level of N-cadherin (Cdh2) in *ATP6AP2-KO* OBs (Fig. 3c, d). Given the reports that N-cadherin-KO impairs OB differentiation, and N-cadherin could interact with LRP6,^38^ we further examined their distribution in control and *ATP6AP2-KD* MC3T3 cells by co-immunofluorescence staining. Interestingly, LRP6 was found to form a complex with N-cadherin and β-catenin at non-adhesive regions of the cell surface (Fig. 7a, b), but not the cell-cell adhesion sites, in OBs (Fig. 7a, c). These cell surface associated LRP6/N-cadherin/β-catenin complexes were un-detectable in *ATP6AP2-KD* OB cells (Fig. 7a, d). Similar with LRP6, N-cadherin’s distribution at the cell surface was reduced, but its localization in LAMP1^+^ lysosomes was increased in *ATP6AP2-KD* MC3T3 cells (Fig. S7a, b). These results suggest that ATP6AP2 may promote both LRP6 and N-cadherin surface targeting, where they interacted with β-catenin, thus form a “β-catenin pool” at the plasma membrane.

### Diminished OB differentiation deficit in *Atp6ap2*^*Ocn-Cre*^ BMSCs by expression of active β-catenin

**Fig. 8:**
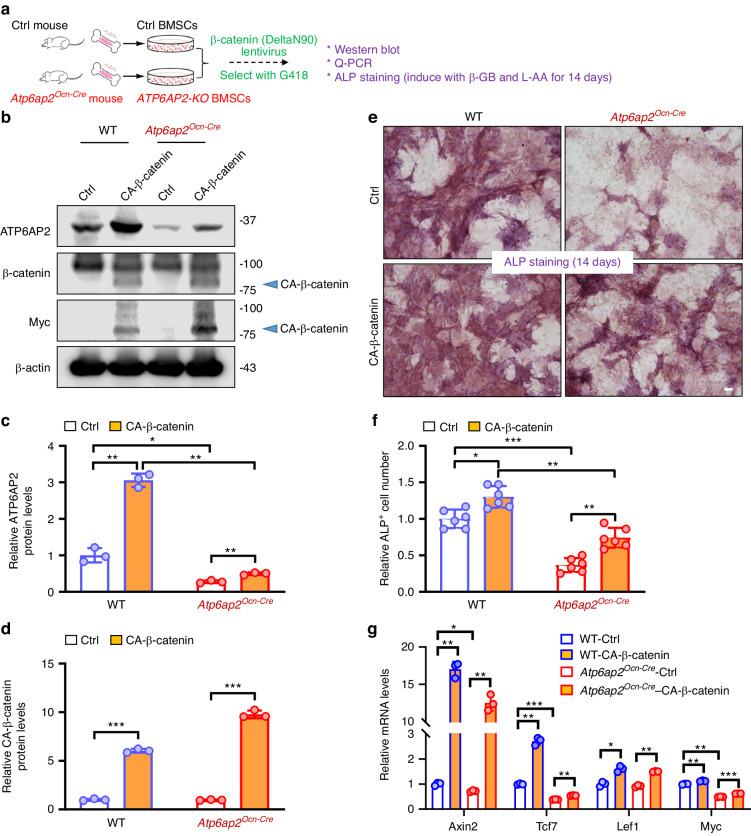
Diminished OB differentiation deficit in *Atp6ap2*^*Ocn-Cre*^ BMSCs by expression of active β-catenin. **a** Experimental strategy. BMSCs derived from 3-month male ctrl or *Atp6ap2*^*Ocn-Cre*^ mice infected with active β-catenin (DeltaN90) lentivirus followed by G418 selection. The cells were then used for western blot, Q-PCR and osteoblast differentiation experiments. **b–d** Western blot of ATP6AP2 and β-catenin in primary cultured BMSCs. β-actin was used as the loading controls. Quantification analyses were shown in (**c**, **d**). **e**, **f** BMSCs were cultured in osteogenic differentiation medium for 14 days (ALP staining). Osteoblast-like cells indicated by ALP (alkaline phosphatase) staining were shown in (**e**). Bar, 20 μm. Quantitative analysis of the ALP cell number was shown in (**f**). **g** Real-time PCR analysis of gene expressions in BMSCs. Data in (**c**), (**d**), (**f**), (**g**) are presented as mean ± SD (*n* = 3 or 6). *P* values obtained by two-way ANOVA followed by Bonferroni post hoc test. *P < 0.05. **P < 0.01. ***P < 0.001, significant difference

To determine whether the impaired Wnt/β-catenin signaling in *Atp6ap2*^*Ocn-Cre*^ BMSCs is responsible for the reduced OB genesis and increased OC-genesis, we expressed the active β-catenin into *ATP6AP2-KO* BMSCs, and examined its effect on OB differentiation. As illustrated in Fig. 8a, a lentivirus encoding β-catenin-DeltaN90 (constitutively active β-catenin, or CA-β-catenin) was generated, which efficiently express CA-β-catenin in BMSCs (Fig. 8b–d). Expression of the CA-β-catenin indeed diminished the OB differentiation deficit in *Atp6ap2*^*Ocn-Cre*^ BMSCs by ALP staining analysis (Fig. 6e, f), and increased expression levels of the -catenin target genes in both ctrl and *ATP6AP2-KO* cells by RT-PCR analysis (Fig. 8g). Note that similar to Wht3a treatment, the CA-β-catenin also significantly increased the mRNA and protein levels of ATP6AP2 (Fig. 8b–d, Fig. S8), supporting the view for ATP6AP2 as a downstream target gene of Wnt3a/β-catenin signaling. Additionally, expression of the CA-β-catenin induced the expression of OPG thus reduced the ratio of RANKl/OPG in both ctrl and *ATP6AP2-KO* BMSCs (Fig. S8d). These results thus support the view for the impaired Wnt3a/β-catenin signaling in *ATP6AP2-KO* BMSCs to be responsible for the OB-differentiation deficit, and implicating the increased RANKL/OPG ratio in *ATP6AP2-KO* BMSCs to be involved in the elevated osteoclast genesis and bone resorption.

## Discussion

**Fig. 9:**
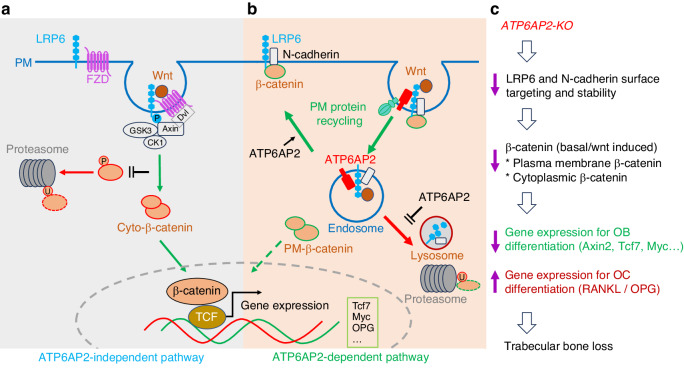
Illustration of a working model. **a–c** Illustration of a working model in which ATP6AP2 deficiency in OB-lineage cells reduces cell surface LRP6 distribution, and thus the cellular response to Wnts stimulation. It also reduces the surface β-catenin levels that binds to LRP6 and N-cadherin, and thus decreasing the basal levels of LRP6/β-catenin proteins. Via β-catenin, ATP6AP2 proteins OBs differentiation and prevents OC genesis and activation, and then maintain the trabecular bone-mass homeostasis

The present study provides evidence for ATP6AP2 in *Ocn-Cre*^*+*^ OB-lineage cells to be critical for both OB and OCs differentiation, and thus preventing the development of osteoporosis. This study also uncovers ATP6AP2’s functions in a cell type specific manner: promoting OBs differentiation and trabecular bone formation likely by stabilizing LRP6/β-catenin and increasing Wnt/β-catenin signaling in trabecular BMSCs or OBs, but not osteocytes. Further mechanistic studies lead to a working model that is depicted in Fig. 9. In this model, ATP6AP2 interacts with LRP6 in BMSCs/OBs, promotes the cell surface targeting of LRP6 and N-cadherin and their associated β-catenin, and prevents them from being degraded by lysosomes and ubiquitin mediated proteosomes, thereby maintaining the basal level of β-catenin and promoting Wnt/β-catenin signaling and the expression of its downstream genes to induce bone formation and inhibit bone resorption.

The canonical Wnt pathway, involves the nuclear translocation of β-catenin and activation of target genes, plays an important role in the development and self-renewal of tissues in mammals. In the absence of Wnts, β-catenin is degraded by the “destruction complexes” in the cytoplasm, which include AXIN, APC, GSK-3β, CK1 and β-trcp. When Wnts are recognized by Fzds and LRP5/6 in the cell membrane, AXIN will bind to phosphorylated LRP5/6.^43^ The destruction complex is believed to be recruited to the cell membrane and thus losing its ability to phosphorylate β-catenin to undergo ubiquitin mediated degradation. Unexpectedly, ATP6AP2 is not required for Wnt3a suppressed β-catenin phorphorylation (Fig. 6), suggesting additional mechanism(s) involved in ATP6AP2 stabilizing β-catenin. For example, Wnt3a decreased ubiquitin-conjugated β-catenin in ctrl, but not *ATP6AP2-KD* cells (Fig. S5), suggesting a role of ATP6AP2 in suppressing β-catenin’s ubiquitination or ubiquitin mediated proteasome degradation. In addition, further cellular studies suggest that ATP6AP2 is required for LRP6-β-catenin protein complex distribution at the cell surface or endosomes under basal condition; and β-catenin nuclear distribution in response to Wnt3a stimulation (Fig. 6). This appears to be a different route to stabilize β-catenin from the Wnt regulation of the destruction complexes in the cytoplasm. In line with above model, many studies have shown that LRP6 endocytosis is essential for Wnt/β-catenin signaling. Blitzer and Nusse have reported that Wnt proteins are rapidly endocytosed by a clathrin- and dynamin-mediated process; and interfering the endocytosis actually blocks Wnt signaling at the level of β-catenin accumulation and target gene expression.^44^ LRP6 is also reported to be internalized with caveolin and that the components of this endocytic pathway are required not only for Wnt3a-induced internalization of LRP6 but also for accumulation of β-catenin.^45^ Caveolin acts on Axin to inhibit the binding of β-catenin to Axin in the process of the transport of the LRP6-containing vesicles to the early endosomes, thereby leading to the accumulation of β-catenin.^45^ These results suggests that a necessary component of Wnt signaling occurs in a subcellular compartment distinct from the plasma membrane and highlights the essential role of LRP6 endocytosis for Wnt/β-catenin signaling.

ATP6AP2 is known to play important roles in multiple signaling pathways and in multiple organs.^9^ In addition to function as a PRR, inducing the conversion of angiotensinogen to angiotensin I and regulating blood pressure,^12^ it is a critical regulator of Wnt/β-catenin signaling and V-ATPase by its interaction with Wnt receptors (e.g., LRP6 and frizzled) and V-ATPase membrane proteins, respectively.^16,17^ It is reported that ATP6AP2 links the V-ATPase, a proton pump that can influence endocytosis by acidification of vesicles, to the Wnt receptor protein LRP6, allowing acidification in the vicinity of the activated receptor, for phosphorylation of LRP6 and subsequent signaling.^16,17^ In line with these reports, we found that ATP6AP2 interacts with LRP6 and regulates LRP6 trafficking (Fig. S6). However, we found that ATP6AP2 stabilizes LRP6, likely by promoting LRP6 targeting to the cellular surface and endosomes, and thus preventing it entering lysosomal compartment for its degradation (Fig. S6). In addition, the cellular surface targeting of N-cadherin, a protein controls cell-cell adhesion, was also regulated by ATP6AP2. We detected a “β-catenin pool” at the plasma membrane of OBs, where it co-located with LRP6 and N-cadherin, which was detectable in control, but not *ATP6AP2-KD* OB cells (Fig. 7). This result is consisted with the literature that N-cadherin could interact with LRP6.^38^ Upon Wnt3a treatment, the cell surface associated LRP6/N-cadherin/β-catenin complex reduced, and the nuclear β-catenin increased in control, but not *ATP6AP2-KD* OBs, suggesting a role of ATP6AP2 in regulating the LRP6-N-cadherin-β-catenin protein complex trafficking and stability, implicating this event to underlie ATP6AP2 stabilization of LRP6/N-cadherin/β-catenin under both basal and Wnt3a stimulated conditions.

The ATP6AP2 regulation of LRP6/β-catenin protein stability occurred in BMSCs or OBs but not Ocys (Figs. 3, 4). In line with these results, examining Wnt/β-catenin signaling in mice showed selective reductions of Axin2-β-gal, (a reporter of Wnt/β-catenin signaling pathway) and β-catenin in the trabecular, but not cortical, bone cells (Fig. 4). Such a cell type dependent regulation of Wnt/LRP6/β-catenin signaling by ATP6AP2 may be due to the differential expression levels of Wnt receptors among BMSCs, OBs and Ocys. Mammals have 2 LRP5/6 co-receptors but 19 Wnts and 10 Fzds receptors.^46^ It is of interest to note Nabhan et al.’ report that different stem cell types in lung alveoli express distinct Fzds receptors, which respond to Wnt ligands in a distinctive manner.^46^ In addition, in our study, although the cell surface levels of both LRP5 and LRP6 were significantly lower in *ATP6AP2-KO* OBs (Fig. 3), the total level of LRP5 was selectively reduced in *ATP6AP2-KO* osteocytes, but not BMSCs (Fig. 4) or OBs (Fig. 3e, f); and the total level of LRP6 was decreased in *ATP6AP2-KO* BMSCs or OBs, but not osteocytes (Figs. 3–4). These results implicate a differential regulation of LRP5 and LRP6 by ATP6AP2 in a cell subtype selective manner, and suggest that ATP6AP2 may regulate LRP6 protein trafficking predominantly in the early stage of OB-lineage cells (e.g., BMSCs), but regulates LRP5 in the later stage (e.g.,osteocytes). This view is also in line with the reports that LRP6 plays a more critical role than that of LRP5 in Wnt3a signaling for OB differentiation.^47,48^ Notice that the study by Riddle et al., has shown that *Lrp6-CKO* mice exhibit earlier (4 weeks) deficit in trabecular bone loss than that of *Lrp5-CKO* mice.^47^ Our results that *ATP6AP2-CKO* in the OB-lineage cells displayed trabecular bone loss as early as 4 weeks (Fig. 1), similar to that of *Lrp6-CKO* mice. However, in contrast with *Lrp5/Lrp6-CKO* or *β-catenin-CKO* mouse’ cortical bone phenotype,^49,50^ *ATP6AP2-CKO* mice showed increased cortical bone mass, suggesting that ATP6AP2 may regulate cortical bone remodeling in a LRP5/6 or wnt/β-catenin independent manner. In light of our proteomics analysis of membrane proteins in control and *ATP6AP2-CKO* OBs, in addition to LRP6, *ATP6AP2-KO* affected multiple membrane proteins’ surface distribution (Fig. 3), including MMP14, also called MT1-MMP, a protein belonging to the matrix metalloproteinase family that is critical for the breakdown of extracellular matrix. We thus speculate that MMP14 may underlie ATP6AP2 regulation of cortical bone phenotype.^51^ Here, our results suggest that LRP6, which is expressed in different levels between OBs and osteocytes, may underlie ATP6AP2’s differential regulation of Wnt signaling. It would be of interest to further test this view in future.

Functionally, the cell type dependent regulation of Wnt/LRP6/β-catenin signaling by ATP6AP2 may underlie ATP6AP2’s selective positive effect in OBs of trabecular bone, but not osteocytes of the cortical bone. In line with this view was our result that expression of active β-catenin in ATP6AP2 mutant BMSCs fully restored the OB differentiation (Fig. 8). This function is likely due to nuclear β-catenin regulated target gene expressions (Fig. 8g).^1,37,52^

In addition to the OB mediated bone formation, ATP6AP2 in the OB-lineage also regulates OC-mediated bone resorption, which is evidenced by increased OC formation and bone resorption in the *Atp6ap2*^*Ocn-Cre*^ mice (Fig. S2). Two mechanisms may underlie the osteoblastic ATP6AP2 regulation of OC genesis. First, ATP6AP2-deficiency in BMSCs/OBs impaired the expression of OPG, thus increased the ratio of RANKL over OPG, and thus promoting OC differentiation from *ATP6AP2-KO* BMMs (Fig. S2 and Fig. S4). Second, we have previously found that ATP6AP2/V-ATPase driven ATP release from OBs could increase the production of the ATP derivative adenosine, which activates A_2A_R signaling in BMMs and reduces receptor activator of nuclear factor κB (RANK)–mediated osteoclastogenesis.^2^

The V-ATPase consists a pore domain (V0 sector) and an ATP hydrolysis domain (V1 sector). V1 sector contains 8 different types of subunits (A-H) and is responsible for ATP hydrolysis, while V0 sector contains 7 different subunits including a, c, c”, d, e, accessory subunit ATP6AP1 (Ac45), and M8-9 (a cleaved C-terminal fragment of ATP6AP2).^53,54^ In humans, ATP6AP1 and ATP6AP2 genes are located on different arms of the X chromosome. ATP6AP2 is highly conserved among species, while ATP6AP1 is poorly conserved.^54^ Like ATP6AP2, ATP6AP1 is also a substrate of furin, and the cleavage of ATP6AP1 by furin has been shown to be required for intragranular acidification.^55^ However, comparing with ATP6AP2, ATP6AP1 has its own features. As the accessory subunit of the V-ATPase, ATP6AP2 binds to the V0 subunits, c and d, while ATP6AP1 binds to the V0 subunits, a3, c, c”, and d. In addition, deletion of the cytosolic terminus of ATP6AP1 altered its binding proximity with a3, c’, and d, thus impair bone resorption in osteoclasts.^56^ Interestingly, knockdown ATP6AP1 in osteoclast not only impairs intracellular acidification and endocytosis, but also result in reduced fusion capacity of osteoclastic precursor cells,^57^ which are different from those in *ATP6AP2 KO* osteoclasts (un-published data). In addition to bone cells, the brain specific Ac45-related protein (Ac45RP), a paralog of the Ac45 protein, also interacts with the V-ATPase V0 and colocalizes with V0 in unconventional, but not synaptic, secretory vesicles of extending neurites, then drives neuronal outgrowth.^58^ Ablation of ATP6AP2 has been reported to selectively suppress expression of the V0 subunits of V-ATPase, resulting in deacidification of the intracellular vesicles.^15^ In line with these reports, we have found an impaired vesicle acidification in *ATP6AP2 KO* osteoblasts, which is critical for vesicular ATP release.^59^ These results suggest that ATP6AP1 and ATP6AP2 may have different functions. The subunits of V-ATPase are ubiquitously expressed, some subunits also have tissue or cell-specific distributions, so the phenotypes of V-ATPase-related human diseases vary from the nervous system, skin, muscle (X-linked myopathy with excessive autophagy, XMEA), kidney (Distal renal tubular acidosis, dRTA) to skeleton (osteopetrosis and osteoporosis) and other tissues.^60^

As a subunits of V-ATPase, ATP6AP2 has been reported to contribute to various pathological events/diseases, such as fibrosis, hypertension, acute kidney injury, cardiovascular disease, cancer, obesity, and various other diseases.^9^ Our study demonstrates a critical role of ATP6AP2 in preventing osteoporosis likely by its interaction with LRP6 and increasing LRP6 surface targeting, then promotes Wnt/LRP6/β-catenin signaling selectively in OBs at the trabecular bone region. The relationship between ATP6AP2 regulation of LRP6/β-catenin signaling and v-ATPase remains unclear. We hope to further address this issue in future.

## Materials and methods

### Ethics statement

All experimental procedures were approved by the Institutional Animal Care and Use Committee at Case Western Reserve University (IACUC, 2017-0121 and 2017-0115), according to US National Institutes of Health guidelines.

### Animals and reagents

The *ATP6AP2*^*flox/X*^ mice (kindly provided by Dr. Frederique Yiannikouris, University of Kentucky; and Dr. Genevieve Nguyen, INSERM, France) were crossed with *osteocalcin (Ocn)-Cre* (kindly provided by Dr. Tom Clemens, Johns Hopkins Medical School) transgenic mice to generate OB- selective conditional knockout (CKO) mutant mice, *Atp6ap2*^*Ocn-Cre*^. The mutant mice were in C57BL/6 J mouse background (for more than 6 generations). The Ai9 and *Axin2*^*LacZ*^ mice were purchased from the Jackson Laboratory (Stock No 007909 and 009120), which are in C57BL/6 J background.

Mouse monoclonal antibodies, including β-actin (A1978, Sigma), β-catenin (BD), Ubiquitin (MAB1510, Millipore), LRP6 (sc-25317, Santa Cruz), Rab7 (ab50533, Abcam) and V5 (V8012, Sigma-Aldrich), were used. Rabbit monoclonal antibodies Lrp1 (ab92544, Abcam) and LRP5 (#5731, Cell signaling) were used. Rabbit polyclonal antibody ATP6AP2 (HPA003156, Sigma Aldrich), N-Cadherin (Abcam, ab18203), phospho-β-catenin (Ser33/37/Thr41, #9561, Cell signaling), phospho-β-catenin (Thr41/Ser45, #9565, Cell signaling) and EEA1 (ab2900, Abcam) were used. Rat Lamp1 (1D4B-c, DSHB) antibody was used. Alexa Fluor 488 phalloidin (f-actin, A12379) was purchased from Thermo Fisher. Secondary antibodies were purchased from Jackson ImmunoResearch Laboratories, Inc. Calcein (C0875) was obtained from Sigma-Aldrich. Recombinant protein Wnt3a (315-20) was obtained from Thermo Fisher Scientific. G418 (10131035) was obtained from Thermo Fisher Scientific. Other chemicals and reagents used in this study were of analytical grade.

### Plasmids and lentiviruses

ATP6AP2-V5-FL and ATP6AP2-V5-ICD plasmids were purchased from DNASU (ATP6AP2 in pLX304, HsCD00446844 and HsCD00437681). ATP6AP2-V5-ECD was made by cloning the ECD domain (17-274) of ATP6AP2 into the pLX304 plasmid (KpnI and XbaI). Lrp6-eGFP (plasmid #180143), N-Cadherin-EGFP (plasmid #18870) and pLV-beta-catenin deltaN90 (plasmid #36985) plasmids were purchased from Addgene. Renin Receptor shRNA Lentiviral Particles (shR-PRR) was obtained from Santa Cruz (sc-62935-V). The authenticity of all constructs was verified by DNA sequencing.

The 3rd generation cre-dependent lentiviral system was used to generate the pLV-beta-catenin deltaN90 lentiviral particle. The lentiviral plasmid (7.5 μg) was co-transfected into 293 cells with packaging plasmid pRSV-Rev (5 μg, Addgene #12253), pMDLg/pRRE (2.5 μg, Addgene #12251), and envelope plasmid pMD2.G (0.8 μg, Addgene #12259) to package the lentivirus. The lentivirus was concentrated before use.

### Primary OB/OC-lineage cell cultures

Whole bone marrow cells were flushed from long bones of Ctrl and ATP6AP2-deficient mice and plated on 100 mm culture plates in DMEM containing 1% penicillin/streptomycin (P/S), 10% fetal bovine serum (FBS) for 2 days. For OB-lineage culture, plates with adherent cells were replaced with fresh culture medium every 3 days. After 7 days, passaging cells (BMSCs) by trypsin digestion, 1 × 10^4^/cm^2^ were plated for experiments. For OB differentiation, 10 mmol/L β-glycerophosphate and 50 μmol/L L-ascorbic acid-2-phosphate were added to the culture medium.

The Ctrl/*ATP6AP2-KO*-CA-beta-catenin BMSCs were obtained by infection of primary cultures BMSCs with lentiviral particles encoding control or pLV-beta-catenin (DeltaN90), respectively. After 5-6 days, stable clones expressing the lentivirus were selected via G418 (200 μg/mL) that induces death of the un-transduced cells.

For OC-lineage culture, non-adherent cells were harvested and subjected to Ficoll-Hypaque gradient centrifugation for purification of BMMs. Cells were plated on 100 mm culture dishes in α-MEM containing 10% FBS, 1% P/S and 10 ng/mL recombinant M-CSF. For osteoclastogenesis, 5×10^4^ BMMs were incubated with OC differentiation medium containing 10 ng/mL recombinant M-CSF and 100 ng/mL recombinant RANKL. Mature OC began to form on day 4 to 5 after RANKL treatment. The cells were then subjected to TRAP staining to confirm their OC identity.

Primary OB cultures were prepared from long bones of mice. Briefly, small bone pieces were incubated in collagenase solution to remove all remaining soft tissue and adhering cells, then transfer to 60 mm culture dishes containing DMEM medium supplemented with 10% FBS, 1% penicillin/streptomycin, 10 mmol/L β-glycerophosphate and 50 μmol/L L-ascorbic acid-2-phosphate. Replace culture medium three times per week. Bone cells will start to migrate from the bone chips after 3–5 days. After two weeks, the monolayer is trypsinized by incubating the cells with trypsin solution. OBs were plated on 100 mm tissue culture plates in α-MEM containing 10% fetal bovine serum (FBS), 1% penicillin/streptomycin (P/S).

Primary osteocyte cultures were isolated from mouse long bones as described previously.^61^ In brief, long bones (femur and tibia) were aseptically dissected from skeletally mature 3-month mice. Collagenase solution was prepared as 300 active U/mL collagenase type-IA (Sigma-Aldrich, St. Louis, MO, USA) dissolved in DMEM. EDTA tetrasodium salt dehydrate (EDTA) solution (5 mmol/L, pH = 7.4; Sigma-Aldrich) was prepared in magnesium and calcium-free Dulbecco’s phosphate-buffered solution (DPBS; Mediatech) with 1% BSA (Sigma-Aldrich). All steps of the digestion took place in 5 mL solution in a 6-well plate, on a rotating shaker set to 200 r/min, in a 37 °C and 5% CO_2_ humidified incubator. Following each sequential digestion, the digest solution with suspended cells was removed from the bone pieces and kept. The combined cell suspension solution was spun down at 900 r/min for 5 min, the supernatant was removed from the cell pellet, and cells were resuspended in culture medium and counted.

### Cell line and transfection

MC3T3-E1 cells were maintained in DMEM supplemented with 10% fetal calf serum and 1% penicillin/streptomycin. For transient transfection, MC3T3-E1 cells were plated at a density of 10^6^ cells per 10-cm culture dish and allowed to grow for 12 h before transfection by using Lipofectamine™ 3000 Transfection Kit (L3000, Invitrogen). 48 h after transfection, cells were subjected to immunostaining analysis.

The *ATP6AP2-KD* cell line was obtained by infection of MC3T3-E1 cells with lentiviral particles encoding scramble control or shRNA-ATP6AP2, respectively. In brief, cells were infected with the lentiviral particles for 1 day in polybrene (2 μg/mL) medium. At day 3, the culture medium was removed and replaced with complete medium (without polybrene). After 5–6 days, stable clones expressing the shRNA were selected via puromycin dihydrochloride (5 μg/mL) that induces death of the un-transduced cells.

### Cell lysis, western blot, and immunoprecipitation assay

Cells were lysed in lysis buffer containing 50 mmol/L Tris-HCl (pH7.5), 150 mmol/L NaCl, 1%(v/v) Triton X-100, 0.1% SDS, 0.5% deoxycholate and 1 mmol/L EDTA, supplemented with protease inhibitors (1 μg/mL leupeptin and pepstatin, 2 μg/mL aprotinin and 1 mmol/L PMSF) and phosphatase inhibitors (10 mmol/L NaF and 1 mmol/L Na_3_VO_4_). Whole cell extracts were fractionated by SDS-PAGE and transferred to a nitrocellulose membrane (Bio-Rad). After incubation with 5% milk in TBST (10 mmol/L Tris, 150 mmol/L NaCl, 0.5% Tween 20, pH 8.0) for 1-h, the membrane was incubated with indicated antibodies overnight at 4 °C. Membranes were washed with TBST for three times and incubated with a 1:5 000 dilution of horseradish peroxidase-conjugated anti-mouse or anti-rabbit antibodies for 1-h. Blots were washed with TBST three times and developed with the ECL system (Bio-Rad). For immunoprecipitation assay, cells were washed once with PBS and lysed for 30 min on ice. Cell lysates were precleared with β-catenin antibody overnight at 4 °C and then with protein A/G beads (Santa Cruz, CA, USA) for another 2 h. After incubation, the protein A/G beads were washed four times with the lysis buffer. The lysates and IP samples were subjected to SDS-PAGE followed by western blot using the indicated antibodies.

### Immunofluorescence staining and imaging analysis

For cell immunofluorescence staining, cells on coverslips were fixed with 4% paraformaldehyde at room temperature for 20 min. Cells were permeabilized with 0.2% Triton X-100 for 8 min, and then subjected to co-immunostaining analysis using indicated antibodies. Stained cells were washed 3 times with PBS and mounted with VECTASHIELD (H-1500; Vector Laboratories) and imaged by Confocal Microscope at room temperature. Fluorescent quantification was performed using ZEN software according to the manufacturer’s instructions (Carl Zeiss).

### Micro-computed tomography (μCT)

The μCT analyses were carried out as described previously.^59,62^ Excised femurs from mice were scanned using the Scanco µCT40 desktop cone-beam micro-CT scanner (Scanco Medical AG, Brüttisellen, Switzerland using µCT Tomography v5.44). Scans were automatically reconstructed into 2-D slices and all slices were analyzed using the µCT Evaluation Program (v.6.5-2, Scanco Medical). The femur was placed inverted in a 12 mm diameter scanning holder and scanned at the following settings: 12 µm resolution, 55 kVp, 145 µA with an integration time of 200 ms. For the cortical analysis, the bone was scanned at the midshaft of the bone for a scan of 25 slices. The region of interest (ROI) was drawn on every slice and fitted to the outside of the cortical bone, to include all the bone and marrow. The threshold for cortical bone was set at 329. The 3-D reconstruction (µCT Ray v3.8) was performed using all the outlined slices. Data were obtained on bone volume (BV), total volume (TV), BV/TV, bone density and cortical thickness. For the trabecular bone, the scan was started at the growth plate and consisted of 211 slices. The region of interest was outlined starting below the growth plate (for the femurs from 1-month mice) and where the condyles ended (for the femurs from older mice). 100 slices were outlined from this point, on the inside of the cortical bone, enclosing only the trabecular bone and marrow. Trabecular bone was thresholded at 245 and the 3-D analysis performed on the 100 slices. Data were obtained on bone volume, density, total volume, trabecular number, thickness and separation.

### Bone histomorphometric analysis

Bone histomorphometric analyses were carried out as previously described.^63^ For X-gal staining, fresh mouse femurs were fixed with 0.2% gluteraldehyde at 4 °C for 5 days, then decalcified in 14% EDTA for 10 days. The samples were place in OCT for cryosection. After cutting, femur sections were washed in PBS and incubated with X-gal staining solution [1 mg/mL X-gal, 5 mmol/L K_3_Fe(CN)_6_, 5 mmol/L K_4_Fe(CN)_6_, 2 mmol/L MgCl_2_ in PBS] at 37 °C overnight. Following several washes in PBS, then counterstained with 0.2% eosin. Slides were mounted with the aqueous mounting medium before taken the images.

### Dynamic bone histomorphometry to measure the rate of bone formation in vivo

Briefly, mice (P30) were injected (intraperitoneally) with fluorochrome-labeled calcein green (10 mg/kg, Sigma–Aldrich) twice (5 days interval). The mice were sacrificed 2 d after the second injection. The femurs were fixed in 70% (vol/vol) ethanol overnight, embedded in methyl methacrylate, and sectioned at 7–10 μm. Images were obtained using Zeiss LSM 800 fluorescence microscope. The mineral apposition rate (MAR) in μm/d and bone formation rate (BFR) [BFR = MAR × MS (mineral surface) / BS (bone surface)] were calculated from fluorochrome double labels at the trabecular bone, periosteum and endosteum surfaces.

### Measurements of serum levels of osteocalcin and PYD

Blood samples were collected and allowed to clot for 30 min and centrifuged for 10 min at 3 000 r/min. Serum was frozen at −80 °C until use. Osteocalcin and PYD were measured in duplicate with osteocalcin Elisa kit (Biomedical Technologies, Inc.) and MicroVue Serum PYD Elisa kit (Quidel Corporation) as described previously.^2^ Concentrations were obtained by comparing readings against standard curves.

### RNA isolation and real time-PCR

Total RNA was isolated by Trizol extraction (Invitrogen, Carlsbad, CA, USA). Q-PCR was performed by using Quantitect SYBR Green PCR Kit (Bio-Rad) with a Real-Time PCR System (Opticon Monitor 3). Runx2 primers (5ʹ-TGACATCCCCATCCATCCAC-3ʹ and 5ʹ-AGAAGTCAGAGGTGGCAGTG-3ʹ), Osx primers (5ʹ-CACCAGGTCCAGGCAACA-3ʹ and 5ʹ-GAGCAAAGTCAGATGGGTAAGT-3ʹ), RANKL primers (5ʹ-ATCCCATCGGGTTCCCATAA-3ʹ and 5ʹ-TCCGTTGCTTAACGTCATGTTAG-3ʹ), OPG primers(5ʹ-GGCCTGATGTATGCCCTCAA-3ʹ and 5ʹ-GTGCAGGAACCTCATGGTCTTC-3ʹ), Lrp5 primers (5ʹ-CACGGGTGTCAAAGAGGC-3ʹ and 5ʹ-CCATTCCTTCAGGGTAGTCG-3ʹ), Lrp6 primers(5ʹ-AATGGCGATGCGAACTGC-3ʹ and 5ʹ-TGGTGGCTTGTGGTGCTG-3ʹ), Axin2 primers (5ʹ-ACCGTGGTTGGCTTGTCC-3ʹ and 5ʹ-CAGTGCGTCGCTGGATAA-3ʹ), Tcf7 primers(5ʹ-TAACTACGGAAAGAAGAAGAGG-3ʹ and 5ʹ-TACCGAATGCATTTCTTTTTCCTCC-3ʹ), Lef1 primers (5ʹ-AATAAAGTGCCCGTGGTG-3ʹ and 5ʹ-ATGCCTTGCTTGGAGTTG-3ʹ), Myc primers(5ʹ-GTCGCTACGTCCTTCTCCC-3ʹ and 5ʹ-TCCTCCAAGTAACTCGGTCA-3ʹ), and β-actin primers (5ʹ-AGGTCATCACTATTGGCAACGA-3ʹ and 5ʹ-CATGGATGCCACAGGATTCC-3ʹ) were used.

### Cell surface biotinylation and proteomic analysis

Cultured OBs were incubated with Sulfo-NHS-biotin (ThermoFisher Scientific) in PBS for 1 h at 4 °C, and treated with 10 mmol/L glycine for 20 min at 4 °C to terminate the crosslinking reaction. After rinse with PBS, cells were lysed in RIPA buffer. Biotinylated proteins were precipitated overnight with avidin agarose beads (Pierce). Finally, beads were washed 3 times with PBS, and 1X SDS loading buffer was added to collect biotinylated surface proteins.

For proteomic assay, biotinylated surface proteins were separated by SDS-Page gel and submitted for LC-MS/MS analysis. The LC-MS system was a ThermoScientific Orbitrap Elite mass spectrometer system. The HPLC column was a Dionex 15 cm×75 μm id Acclaim Pepmap C18, 2μm, 100 Å reversed- phase capillary chromatography column. 5 μL volumes of the extract were injected and the peptides eluted from the column by an acetonitrile/0.1% formic acid gradient at a flow rate of 0.3 μL/min were introduced into the source of the mass spectrometer online. The microelectrospray ion source is operated at 2.5 kV. The digest was analyzed using the data dependent multitask capability of the instrument acquiring full scan mass spectra to determine peptide molecular weights and product ion spectra to determine amino acid sequence in successive instrument scans. The data were analyzed by using all CID/HCD spectra collected in the experiment to search the mouse UniProtKB database with the search Sequest. The protein and peptide identifications were validated with the program scaffold.

### Statistical analysis

All data were expressed as mean ± SD. For in vivo studies, 4–6 mice per genotype per assay were used. For in vitro cell biological and biochemical studies, each experiment was repeated 3 times. Data were analyzed by Student *t* test, two-way ANOVA and post-hoc test (GraphPad Prism 8). The significance level was set at *P* < 0.05.

### Supplementary information

Supplementary Figure Legends

Figure S1

Figure S2

Figure S3

Figure S4

Figure S5

Figure S6

Figure S7

Figure S8
