# Supplementary material for: ATP6AP2, a regulator of LRP6/β-catenin protein trafficking, promotes Wnt/β-catenin signaling and bone formation in a cell type dependent manner
Source: Bone Res. 2024 May 29;12:33. doi: 10.1038/s41413-024-00335-7 (PMC11137048; doi:10.1038/s41413-024-00335-7)
Supplement: Supplementary file 1 — Supplementary Figure Legends [file 41413_2024_335_MOESM1_ESM.docx]

**SUPPLEMENTARY INFORMATION**

**Fig S1. Generation of OB-selective ATP6AP2 KO mice.**

**(a)** Strategy to cleave exon 2 of *Atp6ap2* flanked by loxP sites in the *Atp6ap2^flox^* allele. As *Atp6ap2* is on the X chromosome, *Atp6ap2^flox/X^* female mice were crossed with male osteocalcin (OCN)-Cre transgenic mice to generated OB-selective conditional knockout male mutant mice, Atp6ap2^Ocn-Cre^.

**(b)** Western blotting analysis of ATP6AP2 expression in primary cultured BMSCs and OBs from 3-MO male Ctrl and Atp6ap2^Ocn-Cre^ mice. β-actin was used as the loading controls.

**(c)** Quantification analysis of b.

**(d)** Western blotting analysis of ATP6AP2 expression in primary cultured BMMs and OCs from 3-MO male Ctrl and Atp6ap2^Ocn-Cre^ mice.

**(e)** Quantification analysis of d.

**(f)** Representative photos of male mice with indicated age and genotype.

**(g)** Decreased of body weight in male Atp6ap2^Ocn-Cre^ mice.

Data in (c) and (e) are presented as mean± SD (n=3 independent experiments). *, P< 0.05 as determined by two-way ANOVA with Bonferroni post hoc analysis for multiple comparisons test. Data in (g) are presented as mean± SD (n=6 animals per genotype). P values obtained by unpaired two-tailed t-test. *, P< 0.05. **, P< 0.01.

**Fig S2. Increased bone resorption in Atp6ap2^Ocn-cre^ mice.**

**(a)** ELISA analysis of serum pyridinoline (PYD) levels in 1-MO and 3-MO male ctrl and Atp6ap2^Ocn-Cre^ mice.

**(b-d)** TRAP staining analysis of femur sections from 1-MO and 3-MO male ctrl and Atp6ap2^Ocn-Cre^ mice. Bar, 150 μm. Quantification analysis of TRAP^+^ cell number per unit bone surface area in trabecular bone region or cortical bone region was shown in c and d.

**(e-g)** TRAP staining and bone resorption pit analysis of cultured OCs derived from BMMs from ctrl and Atp6ap2^Ocn-Cre^ mice. Cells were treated with 0.01 μg/mL M-CSF and 100 ng/mL RANKL for 7 d. Bar, 200 μm. For the bone resorption pit assay, OCs were cultured in plates coated with calcium phosphate matrix. Quantification analysis of the number of TRAP^+^ multinucleated cells (MNCs; more than three nuclei) per randomly selected visual field and resorption area were shown in f and g.

Data in (a), (c), (d), (f) and (g) are shown as box plots together with individual data points, and whiskers indicate minimum to maximum (n=6 or 10). P values obtained by unpaired two-tailed t-test. **, P< 0.01. ***, P< 0.001.

**Fig S3. Decreased OB differentiation and mineralization in BMSCs derived from Atp6ap2^Ocn-Cre^ mice.**

**(a)** BMSCs derived from 3-MO male ctrl or Atp6ap2^Ocn-Cre^ mice were cultured in osteogenic differentiation medium for 14 days (ALP staining) or 21 days (Alizarin Red S staining). Osteoblast-like cells indicated by ALP (alkaline phosphatase) staining, and calcification indicated by Alizarin red S staining were shown. Bar, 20 μm.

**(b-c)** Quantitative analysis of the ALP cell number and noduies area (mineralization rate) in A.

**(d)** Relative mRNA expression of Runx2 and Osx during the osteogenic differentiation of BMSCs (1 week).

Data in (b) and (c) are shown as box plots together with individual data points, and whiskers indicate minimum to maximum (n=9 animals per genotype). Data in (d) are presented as mean± SD (n=3 independent experiments). P values obtained by unpaired two-tailed t-test. *, P< 0.05, **, P< 0.01. ***, P< 0.001.

**Fig S4. Up-regulation of ATP6AP2, but down-regulation of LRP5/LRP6 expression, by Wnt3a**

**(a-e)** Real-time PCR analysis of Lrp6, Lrp5, Atp6ap2, Rankl, OPG and Ctnnb1 (beta-catenin) expression in BMSCs derived from the indicated mice with or without wnt3a treatment (100 ng/ml, 12 h).

Data are presented as mean± SD (n=3 independent experiments). P values obtained by two-way ANOVA followed by Bonferroni post hoc test. *, P< 0.05. **, p< 0.01, significant difference.

**Fig S5. Requirement of ATP6AP2 for Wnt3a suppression of β-catenin’s ubiquitination**

**(a-b)** Ctrl and ATP6AP2-KD MC3T3 cells were treated with or without 100 ng/ml Wnt3a for 4 h, followed with 10 μM MG132 for additional 4 h. β-catenin was immunoprecipitated with a β-catenin antibody. Input and immunoprecipitated fractions were analysed by immunoblotting. Quantitative analysis was shown in b.

Data are presented as mean± SD (n=3 independent experiments). P values obtained by two-way ANOVA followed by Bonferroni post hoc test. *, P< 0.05, significant difference.

**Fig S6. Increased LRP6 lysosomal distribution in ATP6AP2-KO MC3T3 cells**

**(a)** Illustration of various deletion mutants of ATP6AP2.

**(b-c)** Co-immunostaining analysis of LRP6-eGFP with various deletion mutants of ATP6AP2 in MC3T3 cells. Representative images are shown in b. Bar, 10 µm. Quantification analysis were shown in C. The co-localization rates of various mutants of ATP6AP2 with LRP6 were determined by the measurement of overlapped signaling (yellow fluorescence) over total GFP^+^ signal.

**(d-e)** Co-immunostaining analysis of LRP6 with EEA1 (a marker for early endosomes), Rab7 (a marker for late endosomes) or Lamp1 (a marker for late endosomes and early lysosomes) in control and ATP6AP2-KD MC3T3 cells. Representative images were shown in d. Images marked with yellow squares were amplified and shown in the right side. Bar, 10 μm. The co-localization index of LRP6 with indicated markers (EEA1, Rab7 and LAMP1) was determined by the measurement of overlapped signaling (yellow fluorescence) over total LRP6-eGFP signal.

**(f-g)** Time-course analysis of LRP6 protein levels after cycloheximide (CHX) treatment. Ctrl or ATP6AP2-KD MC3T3 cell lines were treated with 50 µg/ml CHX for the indicated time. LRP6 protein levels were analyzed by Western blotting. Representative blots are shown in f, and quantification analysis is presented in g.

**(h)** Illustration of a working model in which ATP6AP2 deficiency in OB-lineage cells damage LRP6 trafficking in EEA1^+^ vesicle, consequently reducing cell surface levels of LRP6.

Data in (c) and (e) are shown as box plots together with individual data points, and whiskers indicate minimum to maximum (n = 10). Data in (g) are presented as mean± SD (n=3). P values obtained by unpaired two-tailed t-test. *, P< 0.05. **, P< 0.01. ***, P< 0.001.

**Fig S7. Increased N-cadherin lysosomal distribution in ATP6AP2-KO MC3T3 cells**

**(a-b)** Co-immunostaining analysis of N-cadherin-GFP with EEA1 (a marker for early endosomes), Rab7 (a marker for late endosomes) or Lamp1 (a marker for late endosomes and early lysosomes) in control and ATP6AP2-KD MC3T3 cells. Representative images were shown in a. Images marked with yellow squares were amplified and shown in the right side. Bar, 10 μm. The co-localization index of N-cadherin with indicated markers (EEA1, Rab7 and LAMP1) was determined by the measurement of overlapped signaling (yellow fluorescence) over total N-cadherin-eGFP signal.

Data in (b) is shown as box plots together with individual data points, and whiskers indicate minimum to maximum (n = 10). P values obtained by unpaired two-tailed t-test. **, P< 0.01. ***, P< 0.001.

**Fig S8. Up-regulation of ATP6AP2 and OPG, and down-regulation of Lrp5 and Lrp6 expression in both ctrl and ATP6AP2-KO BMSCs by expression of active β-catenin**

**(a-d)** Real-time PCR analysis of Atp6ap2, Lrp5, Lrp6, Rankl and OPG expression in active beta-catenin expressed BMSCs.

Data are presented as mean± SD (n=3 independent experiments). P values obtained by two-way ANOVA followed by Bonferroni post hoc test. *, P< 0.05. **, p< 0.01. ***, p< 0.001, significant difference.
