# Supplementary figures and images for: ATP6AP2, a regulator of LRP6/β-catenin protein trafficking, promotes Wnt/β-catenin signaling and bone formation in a cell type dependent manner
Source: Bone Res. 2024 May 29;12:33. doi: 10.1038/s41413-024-00335-7 (PMC11137048; doi:10.1038/s41413-024-00335-7)

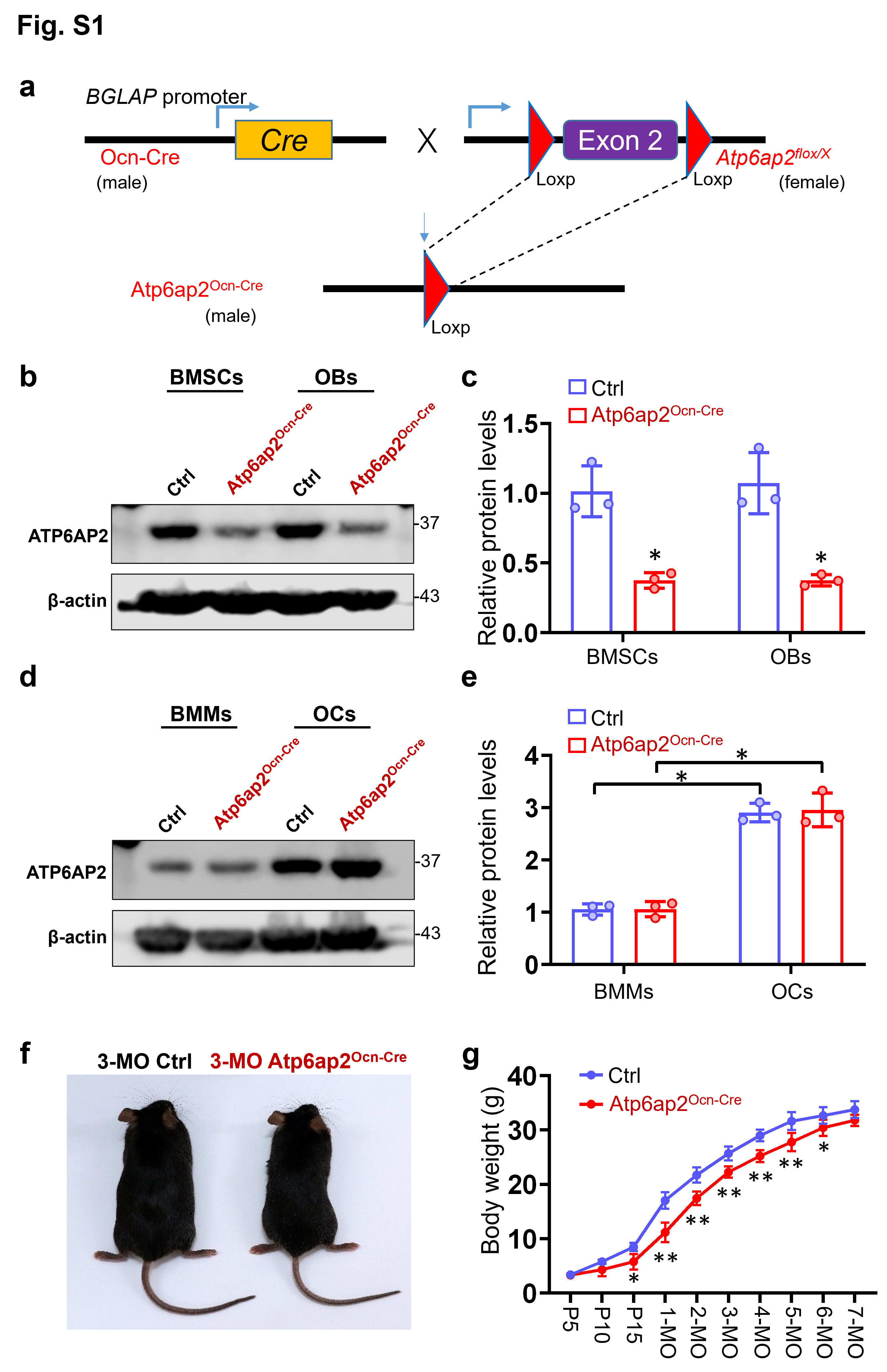

Supplement: Supplementary file 2 — Figure S1 [file 41413_2024_335_MOESM2_ESM.tif]

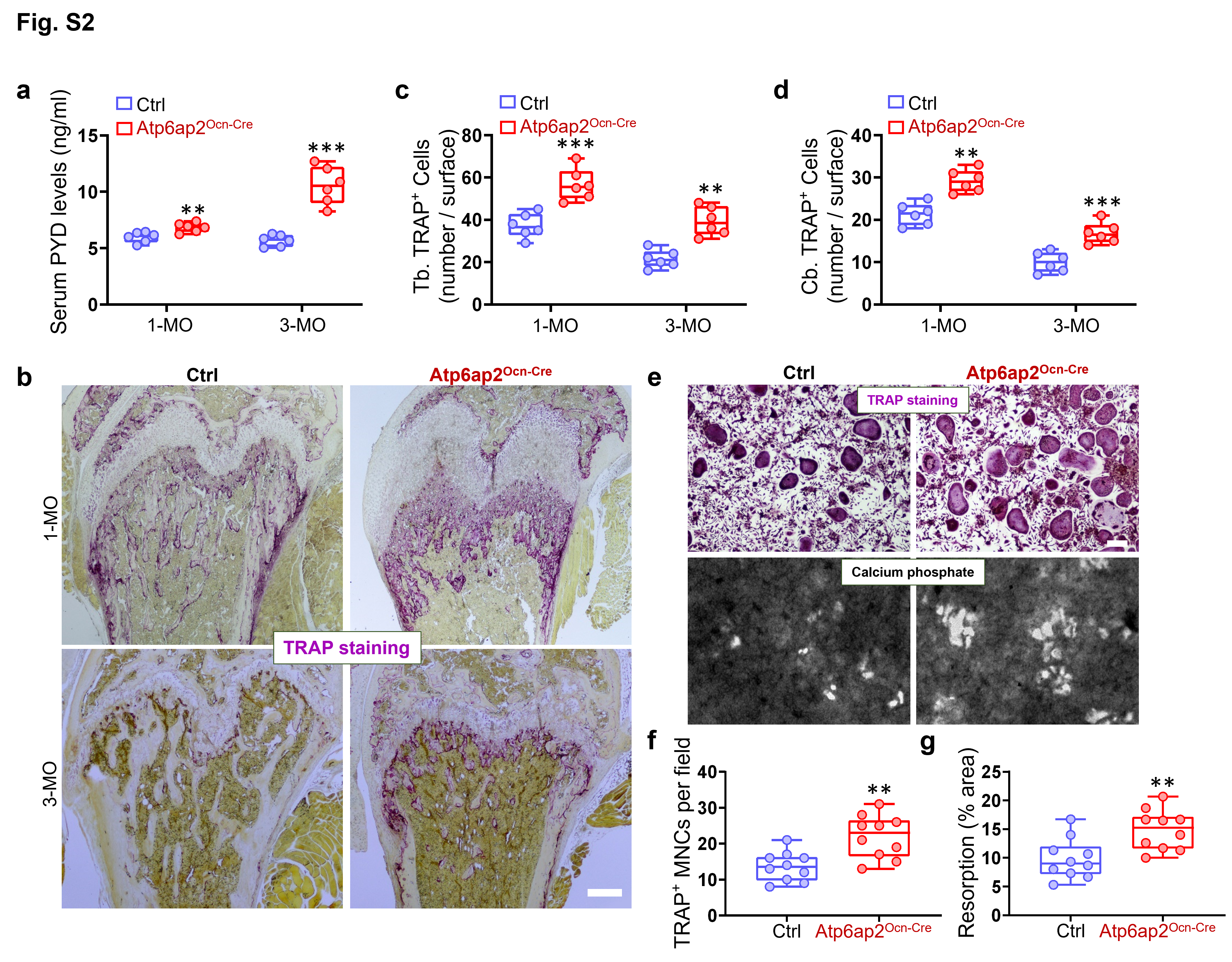

Supplement: Supplementary file 3 — Figure S2 [file 41413_2024_335_MOESM3_ESM.tif]

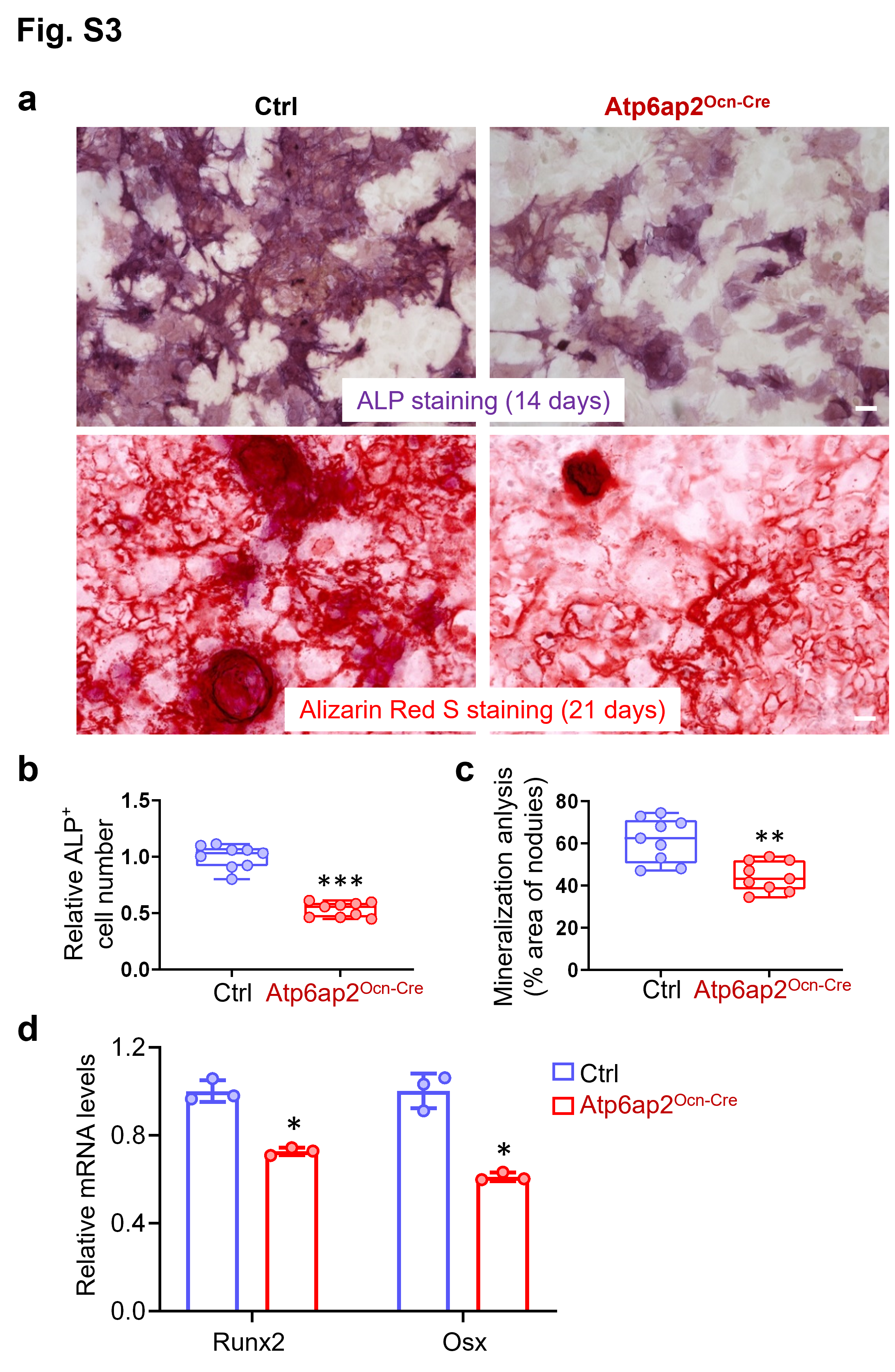

Supplement: Supplementary file 4 — Figure S3 [file 41413_2024_335_MOESM4_ESM.tif]

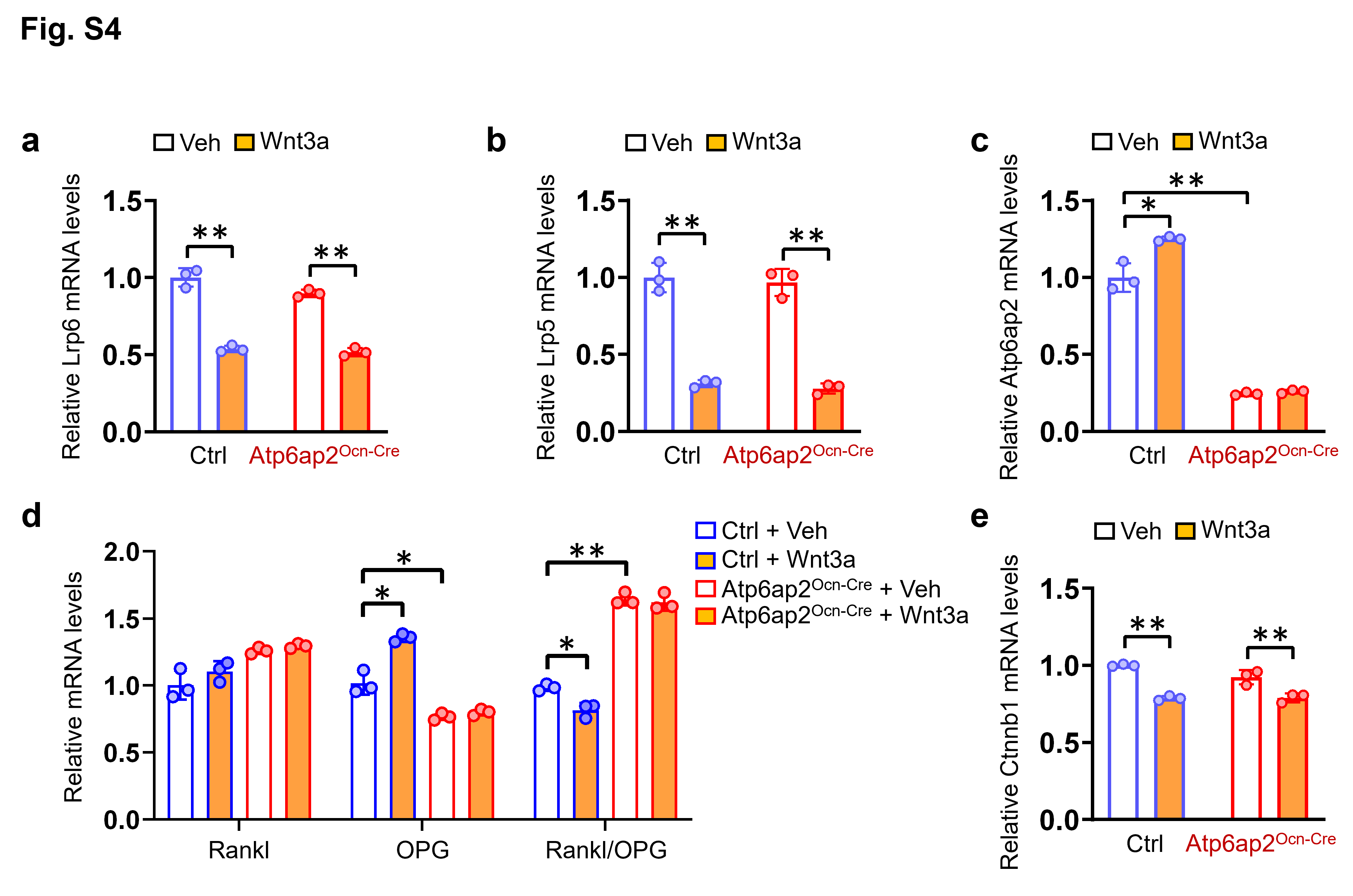

Supplement: Supplementary file 5 — Figure S4 [file 41413_2024_335_MOESM5_ESM.tif]

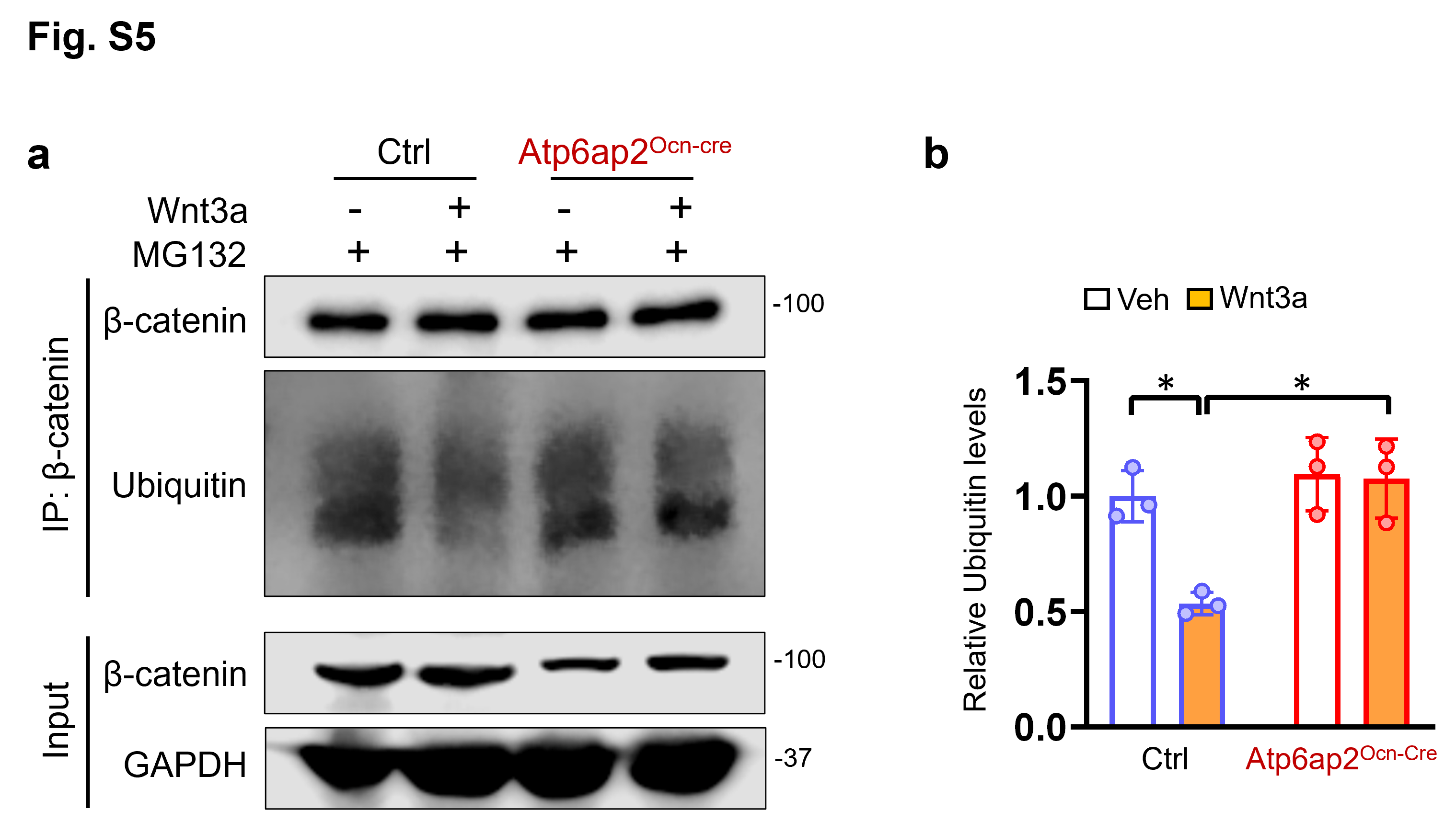

Supplement: Supplementary file 6 — Figure S5 [file 41413_2024_335_MOESM6_ESM.tif]

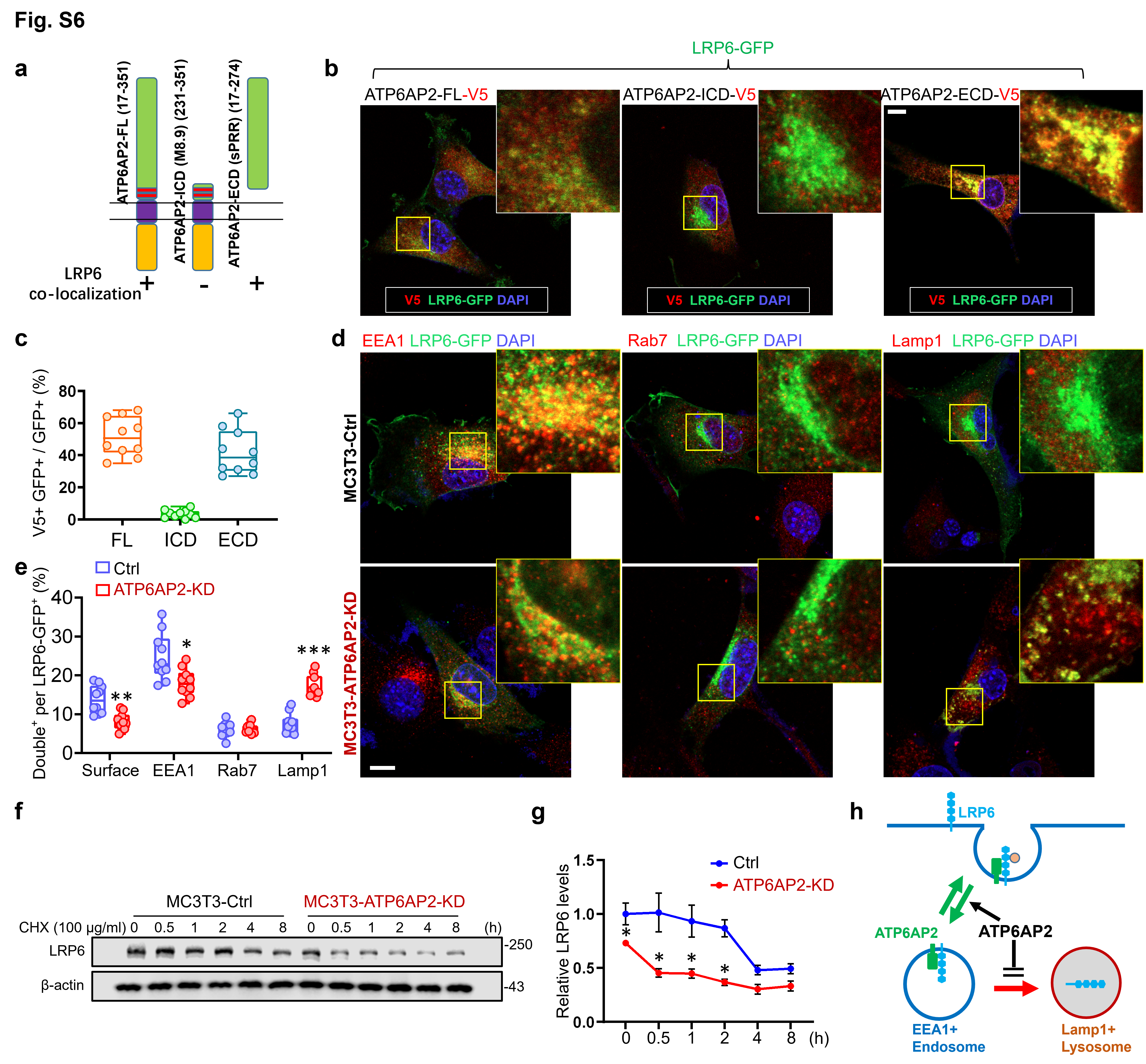

Supplement: Supplementary file 7 — Figure S6 [file 41413_2024_335_MOESM7_ESM.tif]

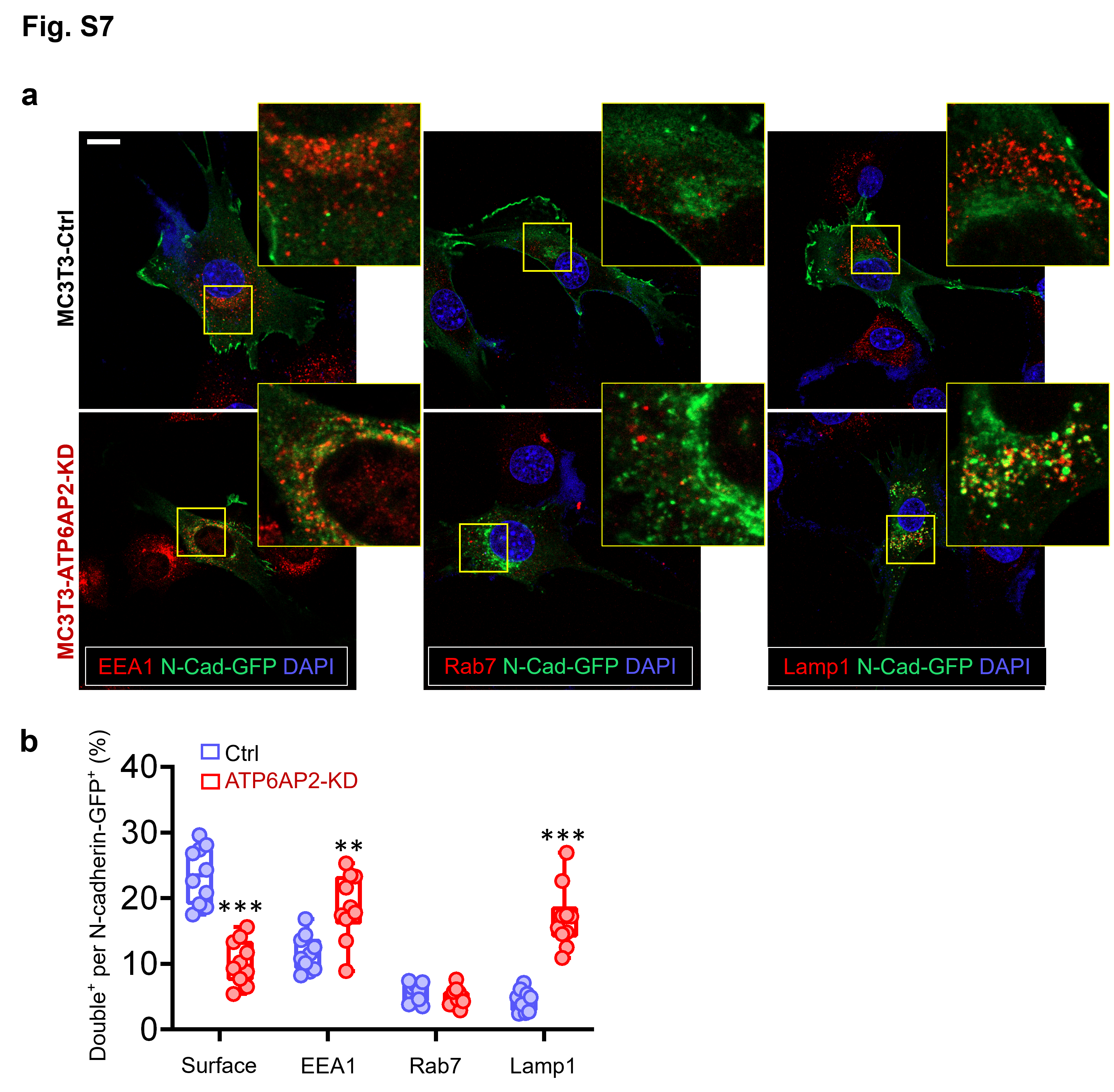

Supplement: Supplementary file 8 — Figure S7 [file 41413_2024_335_MOESM8_ESM.tif]

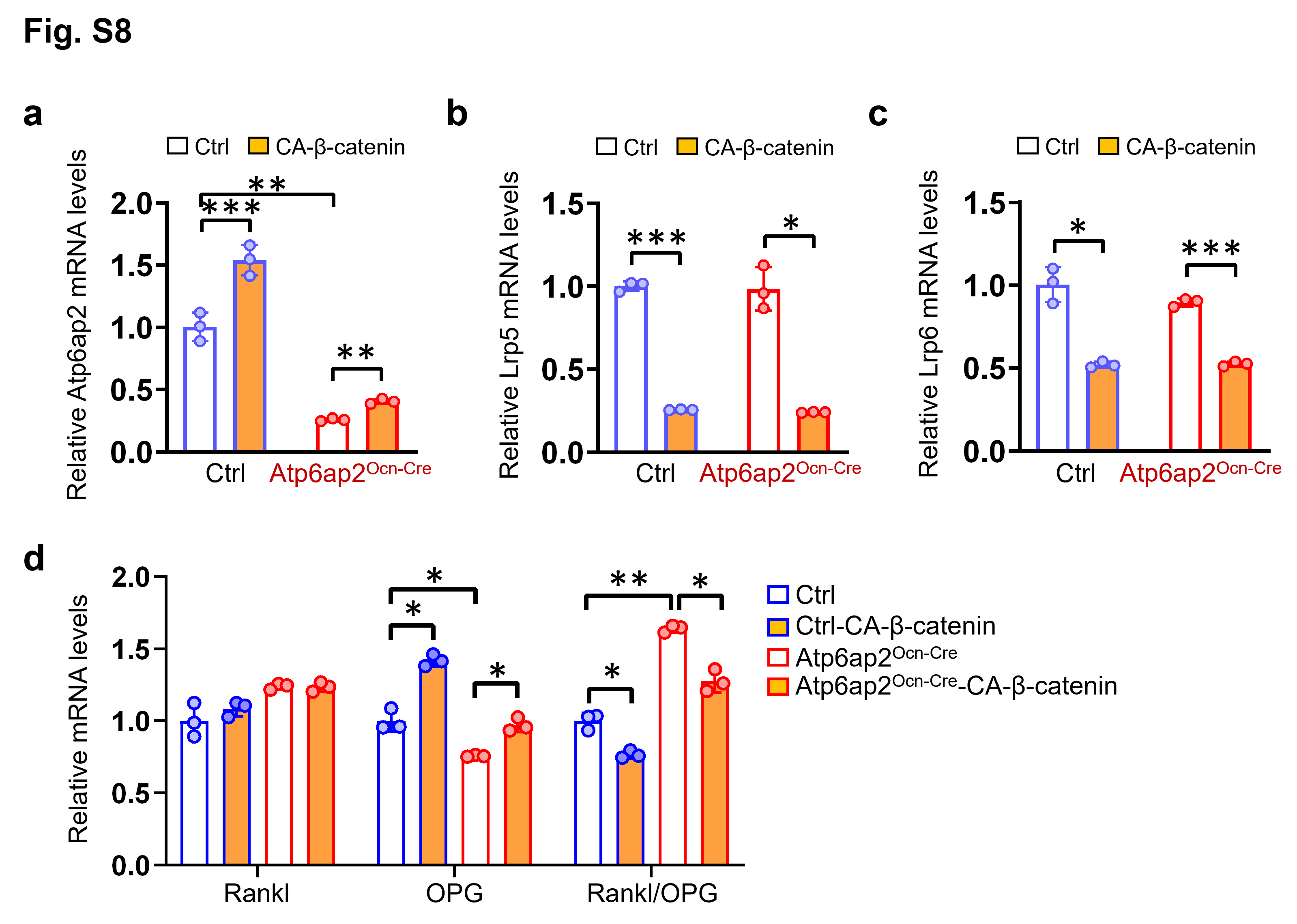

Supplement: Supplementary file 9 — Figure S8 [file 41413_2024_335_MOESM9_ESM.tif]
